# Organic cation transporter 3 (Oct3) is a distinct catecholamines clearance route in adipocytes mediating the beiging of white adipose tissue

**DOI:** 10.1371/journal.pbio.2006571

**Published:** 2019-01-17

**Authors:** Wenxin Song, Qi Luo, Yuping Zhang, Linkang Zhou, Ye Liu, Zhilong Ma, Jianan Guo, Yuedong Huang, Lili Cheng, Ziyi Meng, Zicheng Li, Bin Zhang, Siqi Li, Sook Wah Yee, Hao Fan, Peng Li, Kathleen M. Giacomini, Ligong Chen

**Affiliations:** 1 School of Pharmaceutical Sciences, Tsinghua University, Beijing, China; 2 Department of Bioengineering and Therapeutic Sciences, Schools of Pharmacy and Medicine, University of California, San Francisco, California; 3 Institute for Human Genetics, University of California, San Francisco, California; 4 State Key Laboratory of Membrane Biology, Tsinghua-Peking Center for Life Sciences, School of Life Sciences, Tsinghua University, Beijing, China; 5 Institute of Immunology, School of Medicine, Tsinghua University, Beijing, China; 6 Bioinformatics Institute, Agency for Science, Technology and Research, Singapore; Department of Biological Sciences, National University of Singapore, Singapore; Centre for Computational Biology, DUKE-NUS Medical School, Singapore; 7 Collaborative Innovation Center for Biotherapy, State Key Laboratory of Biotherapy and Cancer Center, West China Hospital, West China Medical School, Sichuan University, Chengdu, China; Harvard School of Public Health, United States of America

## Abstract

Beiging of white adipose tissue (WAT) is a particularly appealing target for therapeutics in the treatment of metabolic diseases through norepinephrine (NE)-mediated signaling pathways. Although previous studies report NE clearance mechanisms via SLC6A2 on sympathetic neurons or proinflammatory macrophages in adipose tissues (ATs), the low catecholamine clearance capacity of SLC6A2 may limit the cleaning efficiency. Here, we report that mouse organic cation transporter 3 (Oct3; Slc22a3) is highly expressed in WAT and displays the greatest uptake rate of NE as a selective non-neural route of NE clearance in white adipocytes, which differs from other known routes such as adjacent neurons or macrophages. We further show that adipocytes express high levels of NE degradation enzymes *Maoa*, *Maob*, and *Comt*, providing the molecular basis on NE clearance by adipocytes together with its reuptake transporter Oct3. Under NE administration, ablation of *Oct3* induces higher body temperature, thermogenesis, and lipolysis compared with littermate controls. After prolonged cold challenge, inguinal WAT (ingWAT) in adipose-specific *Oct3*-deficient mice shows much stronger browning characteristics and significantly elevated expression of thermogenic and mitochondrial biogenesis genes than in littermate controls, and this response involves enhanced β-adrenergic receptor (β-AR)/protein kinase A (PKA)/cyclic adenosine monophosphate (cAMP)-responsive element binding protein (Creb) pathway activation. Glycolytic genes are reprogrammed to significantly higher levels to compensate for the loss of ATP production in adipose-specific *Oct3* knockout (KO) mice, indicating the fundamental role of glucose metabolism during beiging. Inhibition of β-AR largely abolishes the higher lipolytic and thermogenic activities in *Oct3-*deficient ingWAT, indicating the NE overload in the vicinity of adipocytes in *Oct3* KO adipocytes. Of note, reduced functional alleles in human *OCT3* are also identified to be associated with increased basal metabolic rate (BMR). Collectively, our results demonstrate that Oct3 governs β-AR activity as a NE recycling transporter in white adipocytes, offering potential therapeutic applications for metabolic disorders.

## Author summary

Adipose tissues (ATs) can be divided into three distinct types: white fat (or white AT [WAT]), brown fat, and beige fat. Growing evidence suggests that the development of beige fat cells in WAT, also called browning or beiging of WAT, might protect against obesity and improve systemic metabolism. Norepinephrine (NE)-induced β-adrenergic signaling is a major regulator of adaptive thermogenesis (a process of generating heat under conditions of physical activity) and leads to activation of protein kinase A (PKA) and phosphorylation of cyclic adenosine monophosphate (cAMP)-responsive element binding protein (Creb), thereby controlling the expression of thermogenic genes. In this study, we found that the catecholamine transporter, mouse organic cation transporter 3 (Oct3), is highly expressed in WAT, where it mediates NE uptake in the white fat cells in vivo and in vitro. Removing Oct3 in the fat cells leads to enhanced lipid breakdown, increased thermogenesis, and browning of WAT when stimulated by NE or cold exposure via activation of the β-adrenergic receptor (β-AR)/PKA/Creb pathway. In humans, reduced functional alleles of *OCT3* are also associated with increased basal metabolic rate (BMR). Our results indicate that Oct3 is an essential regulator of NE recycling and the beiging of WAT.

## Introduction

Obesity, a disease characterized by excess body fat, is a major risk factor for many human diseases, including type 2 diabetes, cardiovascular disease, and hepatic steatosis [1]. In mammals, fat is stored primarily in adipose tissue (AT), and three distinct types of ATs have been characterized: white AT (WAT), brown AT (BAT), and beige AT [2]. The morphology, function, cellular origins, and molecular features of the three types of ATs are quite distinct [3]. WAT stores nutrients as triglycerides in unilocular adipocytes, which can be used to generate free fatty acids (FFAs) by lipolysis [4]. BAT is the main tissue responsible for thermogenesis, especially when stimulated by cold [5]. After prolonged thermogenic induction, brown-like adipocytes can also be found in WAT, thus named beige or brite adipocytes [3]. The heat produced by BAT or beige AT is indispensable for survival during cold acclimatization. Also, the adaptive thermogenic beige AT may have the potential to counteract obesity and its related disorders [6, 7].

Beiging of WAT can be induced by various stimuli, including cold challenge, bile acids [8, 9], pharmacological agents, and hormones such as norepinephrine (NE) [3, 10]. NE activates β-adrenergic receptor (β-AR), which is coupled to G-proteins, resulting in increases in cyclic adenosine monophosphate (cAMP) concentration in adipocytes that enhance the activity of cAMP-dependent protein kinase A (PKA) [11]. This signal not only leads to lipolysis by phosphorylating hormone sensitive lipase (Hsl) but also by phosphorylating cAMP-responsive element binding protein (CREB), subsequently increasing the expression levels of thermogenic genes (*UCP1*, *PGC1α*, and *DIO2*) to promote nonshivering thermogenesis [11, 12]. The tightly regulated dichotomy of NE release and clearance maintains the balance between NE-induced thermogenesis and excessive NE in the vicinity of adipocytes. Thus, it is critical to elucidate the mechanism of NE clearance in AT. Although previous studies reported NE clearance mechanisms via SLC6A2 on sympathetic neurons [13] or proinflammatory macrophages in AT [14, 15], the low catecholamine clearance capacity of SLC6A2 may limit its cleaning efficiency.

Catecholamines, including NE, epinephrine, serotonin, and dopamine, act as neuromodulators in the central nervous system and as hormones in ATs and blood circulation [16]. Catecholamines are actively cleared in extracellular environment and are translocated into cells by specialized transporters that belong to two distinct transport mechanisms: neuronal transport (Uptake_1_, which is mediated by SLC6A2, SLC6A3, and SLC6A4) and extraneuronal transport (Uptake_2_, which is mediated by organic cation transporters [OCTs]) [17]. Studies have suggested a role of AT in the clearance and metabolism of catecholamines. Indeed, it has been shown that plasma concentrations of epinephrine and NE decrease after passing through AT [18, 19]. In addition, human adipocytes exhibit high expression levels and enzymatic activity of the catecholamine-degrading enzyme monoamine oxidase (MAO) [20]. Transport of catecholamines by adipocytes displays similar characteristics as the Uptake_2_ system and can be inhibited by the OCT3 inhibitor disprocynium 24 [20]. Ayala-Lopez et al. identified that perivascular ATs in male Sprague-Dawley rats had a NE uptake mechanism and could be reduced by norepinephrine transporter (NET) or/and OCT3 inhibitors ex vivo [21]. We reason that extraneuronal monoamine transporter OCT3 is an important route in NE clearance and adaptive thermogenesis in adipocytes complementary to sympathetic neurons or macrophages. In addition, the *SLC22A3* (*OCT3*)*-LPAL2-LPA* gene cluster has been identified as a risk locus for coronary artery disease, implying its potential relationship to lipolysis [22].

In this study, we identified Oct3 as a novel non-neural NE clearance route in adipocytes and demonstrated its role in governing β-AR activity to mediate the beiging of WAT. Adipose-specific *Oct3* knockout (KO) mice under cold acclimatization or NE administration exhibited significantly higher expression levels of genes related to thermogenesis, mitochondria biogenesis, and glycolysis in inguinal WAT (ingWAT) compared with *Oct3*^*fl/fl*^ (Ctrl) mice. Of note, through mining of the Genome-Wide Association Studies (GWAS) Catalog, the database of Genotypes and Phenotypes (dbGAP), and UK BioBank databases, we also identified genetic variants of human *OCT3* associated with higher basal metabolic rate (BMR). These findings suggest that Oct3 regulates catecholamines levels in the vicinity of the β-AR in adipocytes and therefore plays essential roles in the browning of WAT during adaptive thermogenesis. Inhibition of OCT3 may provide distinct therapeutic application by activating energy expenditure pathways.

## Results

### *Oct3* was predominantly expressed in AT and had a preferred uptake of NE

**Fig 1:**
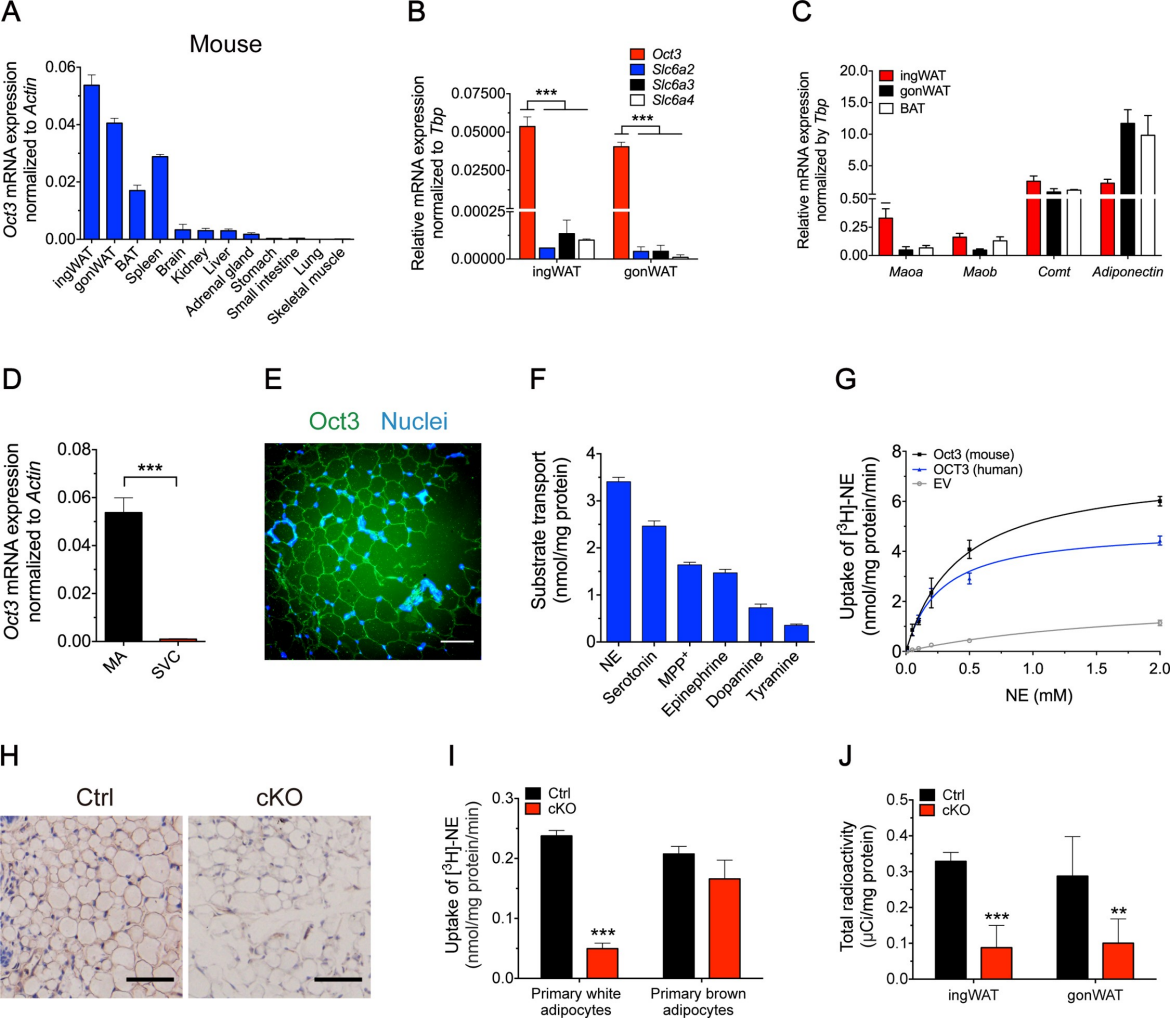
*Oct3* was highly expressed in AT and had the greatest uptake rate of NE. (A) Tissue distribution of mouse *Oct3* mRNA (8-week-old male C57BL/6 mice, *n* = 3). (B) mRNA expression of *Oct3*, *Slc6a2*, *Slc6a3*, and *Slc6a4* in different mouse WATs (*n* = 3). (C) mRNA expression of *Maoa*, *Maob*, *Comt*, and adipocytes abundant gene *Adiponectin* in different mouse WATs (*n* = 3). (D) mRNA expression of *Oct3* in mouse MAs and SVCs of ingWAT (*n* = 3). (E) Oct3 immunofluorescence staining (green) of mouse primary inguinal adipocytes. DAPI (blue), nucleus. Scale bar, 50 μm. (F) Uptake of different monoamines and MPP^+^ in HEK-293 cells stably overexpressing mouse *Oct3*. MPP^+^ was used as a model substrate for functional study of *Oct3*. *n* = 3 experiments. (G) Saturation kinetics of [^3^H]-NE transport in HEK-293 Flp-In cells stably expressing human *OCT3* or mouse *Oct3* (*n* = 3–4). (H) Immunohistochemical staining of Oct3 in ingWAT of Ctrl and adipose-specific *Oct3* KO (cKO) mice. Scale bar, 50 μm. (I) [^3^H]-NE uptake assay in primary white and brown adipocytes from Ctrl and cKO mice. (J) Total radioactivity in ingWAT and gonWAT from Ctrl and cKO mice after [^3^H]-NE injection (*n* = 6). Data in panel B were analyzed by one-way ANOVA followed by Tukey’s test. Data in panels D, I, and J were analyzed by Student *t* test. The numerical data underlying this figure are included in S1 Data. AT, adipose tissue; BAT, brown adipose tissue; cKO, conditional knockout; Ctrl, control; EV, empty vector; gonWAT, gonadal white adipose tissue; HEK, human embryonic kidney; ingWAT, inguinal white adipose tissue; KO, knockout; MA, mature adipocyte; MPP, methyl-4-phenylpyridinium; NE, norepinephrine; Oct3, organic cation transporter 3; SVC, stromal vascular cell; WAT, white adipose tissue.

*Oct3* mRNA levels were examined in several C57BL/6J mouse tissues by real-time PCR. The highest expression levels were found in ingWAT, followed by gonadal WAT (gonWAT) (Fig 1A). In human tissues, *OCT3* had the highest transcript levels in skeletal muscle and liver, followed by ATs (S1A Fig). Compared with transcript levels of other catecholamine transporters *Slc6a2*, *Slc6a3*, and *Slc6a4* in murine WAT, mRNA levels of *Oct3* were much higher (Fig 1B), suggesting that Oct3 is the predominant transporter for catecholamines in WAT. We further showed that adipocytes expressed high levels of NE degradation enzymes *Maoa*, *Maob*, and *Comt*, which was as high as the adipocytes abundant gene *Adiponectin* in ingWAT (Fig 1C), providing the molecular basis on NE clearance by adipocytes itself together with its transporter Oct3. Fractionation of ingWAT showed that *Oct3* was enriched in mature adipocytes rather than in stromal vascular cells (SVCs) (Fig 1D), with *Perilipin* used as a marker for mature adipocytes, as its expression was limited to mature adipocytes and was not found in SVC (S1B Fig). Similar expression patterns of *Oct3* in mature adipocytes and SVC were also observed in gonWAT (S1C and S1D Fig). Immunofluorescence of WAT localized Oct3 to cell membranes of adipocytes (Fig 1E).

To compare the Oct3 uptake activity against its main substrates (NE, serotonin, 1-methyl-4-phenylpyridinium (MPP^+^), epinephrine, dopamine, and tyramine), substrate uptake assays were performed in HEK-293 Flp-In cells stably expressing *Oct3*. NE had the highest uptake activity when cells were incubated with equivalent concentrations of catecholamines (Fig 1F). Human OCT3 and mouse Oct3-overexpressing HEK-293 cells both showed strong NE transport activities compared with empty vector (EV) in vitro, with *K*_*m*_ and *V*_*max*_ of 0.182 ± 0.0275 mM, 3.57 ± 0.174 nmol/mg protein/min (human) and 0.336 ± 0.0726 mM, 5.77 ± 0.448 nmol/mg protein/min (mouse), respectively (Fig 1G). Both *K*_*m*_ and *V*_*max*_ of OCT3 were more than 600 times greater than previously reported values of the Uptake_1_ transporter, SLC6A2 (0.28 ± 0.03 μM; 5.83 ± 0.49 pmol/mg protein/min) [23], consistent with OCT3 being a high-capacity, low-affinity Uptake_2_ transporter of NE. These data suggested an important role of OCT3 in regulating NE concentrations in peripheral tissues including AT, especially when a high concentration of NE existed, such as under NE stimulation or cold challenge.

To further examine the interactions between catecholamines and Oct3, we built a three-dimensional Oct3 homology model based on human glucose transporter 3 (GLUT3) template. Docking of structurally divergent catecholamines (NE, epinephrine, histamine, dopamine, serotonin, and tyramine) indicated that epinephrine (E = −45.7) and NE (E = −43.6) showed the best docking energy scores, resulting from favorable interactions formed between these two bioamines and polar residues in the primary substrate binding site of Oct3 including K215, Q242, E385, and E446 (S1E Fig).

To address the connection between Oct3, catecholamines, and adipocyte metabolism in vivo, we crossed Oct3^fl/fl^ mice with Adiponectin-Cre mice to conditionally delete *Oct3* in adipocytes (S2A Fig). Disruption of *Oct3* was confirmed by immunohistochemistry (Fig 1H) and western blot analysis in ATs (S2B Fig). In addition, no compensational changes were observed in expression levels of the other catecholamine transporters *Slc6a2*, *Slc6a3*, and *Slc6a4* in *Oct3*-deficient ATs compared with controls (S2C Fig). When fed a normal chow diet, adipose-specific *Oct3* KO mice were healthy and viable, with no significant differences in body weight (S2D Fig), metabolic parameters (S2E–S2H Fig), or adipose morphology (S2I Fig) compared with Ctrl mice under room temperature (RT).

To determine NE uptake activity in different adipocytes, we performed [^3^H]-NE uptake assays in primary white and brown adipocytes. When treated with NE at a physiologically relevant concentration (0.20 μM), NE accumulation was significantly reduced (80% reduction) in *Oct3* KO white adipocytes compared with controls, but not in brown adipocytes, suggesting that Oct3 is required for NE uptake in white adipocytes (Fig 1I). Moreover, in vivo NE uptake assay was performed after chemical sympathectomy, which eliminated neural NE uptake in ingWAT (S3A and S3B Fig). *Oct3*-null WAT showed significantly reduced total radioactivity, which reflected the accumulation of NE and its metabolites in adipocytes (73% and 65% reduction in ingWAT and gonWAT, respectively) (Fig 1J). Both the in vitro and in vivo data supported the role of Oct3 as the NE transporter in WAT.

### NE-induced thermogenesis was augmented in adipose-specific *Oct3* KO mice

**Fig 2:**
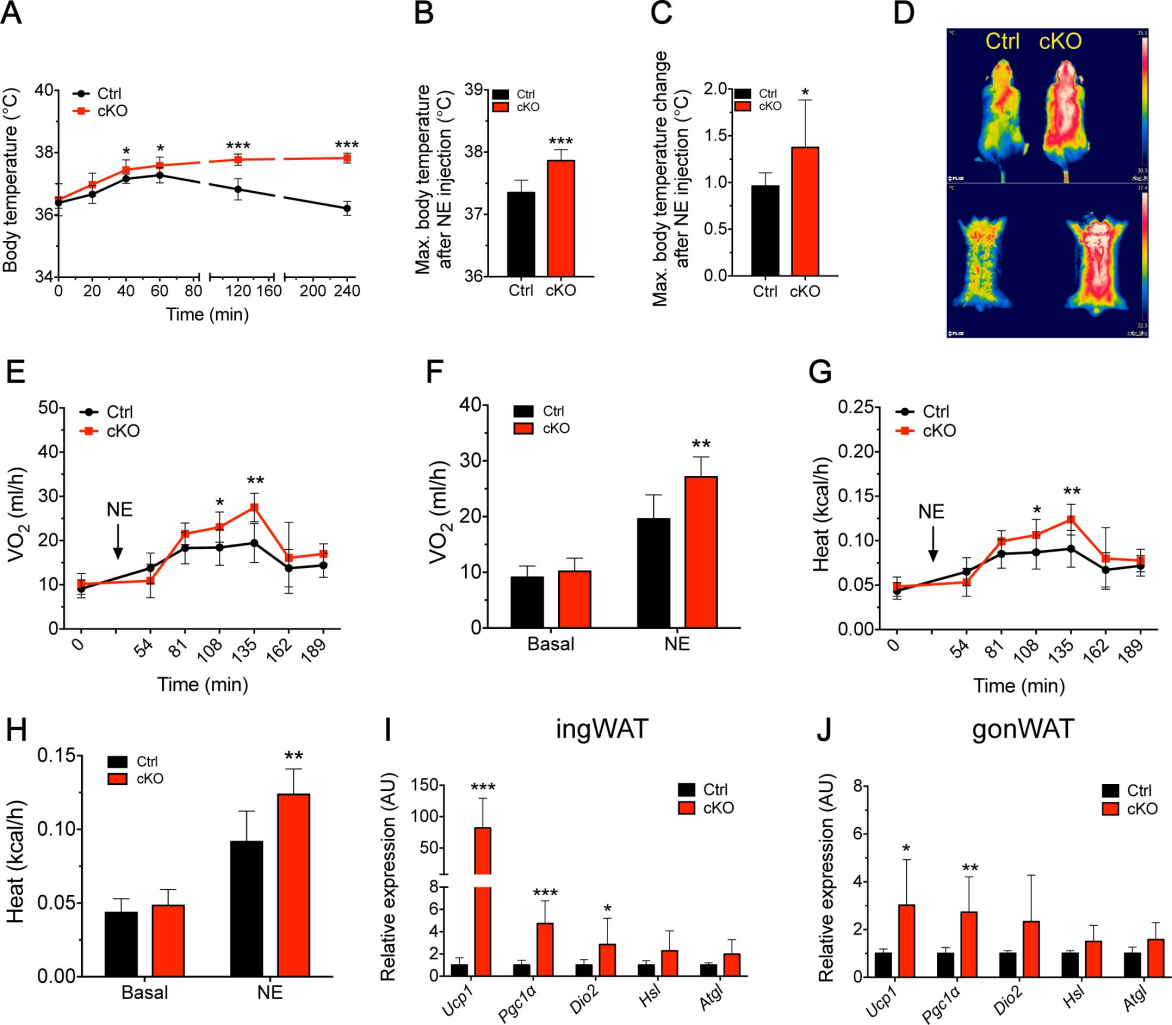
Adipose-specific *Oct3* KO mice exhibited enhanced NE-induced thermogenesis. (A–C) Body temperature parameters of Ctrl and cKO mice after NE injection (s.c., 0.3 mg/kg) at the indicated time point (*n* = 8). (A) Rectal core body temperature; (B) maximal body temperature; (C) maximal body temperature change. (D) Representative thermal images of Ctrl and cKO mice after NE injection. (E–H) Metabolic parameters of Ctrl and cKO mice after NE injection (*n* = 8). (E, F) Relative O_2_ consumption; (G, H) Heat production. (I, J) mRNA expression of thermogenic and lipolytic genes in ingWAT (panel I) and gonWAT (panel J) of Ctrl and cKO mice after NE injection (*n* = 8). Data in A–C and I–J were analyzed by Student *t* test. Data in E–H were analyzed by ANCOVA. The numerical data underlying this figure are included in S1 Data. AU, arbitrary unit; cKO, conditional knockout; Ctrl, control; gonWAT, gonadal white adipose tissue; Hsl, hormone-sensitive lipase; ingWAT, inguinal white adipose tissue; KO, knockout; NE, norepinephrine; Oct3, organic cation transporter 3; s.c., subcutaneous; Ucp1, uncoupling protein 1; VO_2_, oxygen consumption.

Given that fact that NE was a preferential Oct3 substrate, we sought to investigate whether genetic deletion of *Oct3* would influence NE clearance and NE-induced thermogenesis in vivo. After subcutaneous injection of low-dose NE (0.3 mg/kg), adipose-specific *Oct3* KO mice showed significantly higher body temperature (Fig 2A and 2D) and higher maximum body temperature (Fig 2B and 2C) than that of Ctrl mice, as well as prolonged retention of increased body temperature (Fig 2A).

Adipose-specific *Oct3* KO and Ctrl mice that received NE injections both demonstrated marked elevations in both whole-body O_2_ consumption (Fig 2E and 2F) and heat production (Fig 2G and 2H), while these parameters remained consistent between both genotypes under nonintervention conditions (S2E–S2H Fig). The magnitude of NE-induced O_2_ consumption (Fig 2E and 2F) and heat production (Fig 2G and 2H) in adipose-specific *Oct3* KO mice was significantly higher than in the Ctrl littermates. Among the thermogenic markers tested, adipose-specific *Oct3* KO mice treated with NE displayed a nearly 82-fold increase in *Ucp1*, a 5-fold increase in *Ppargc1α* (*Pgc1α)*, and a 3-fold increase in *Dio2*, as well as an increasing trend in *Hsl* and *Atgl* mRNA expression in ingWAT (Fig 2I). *Ucp1*, *Pgc1α*, and *Dio2* were also increased in gonWAT of adipose-specific *Oct3* KO mice (Fig 2J). However, the relative expression of *Ucp1* in gonWAT was far lower than that in ingWAT, suggesting that ingWAT might be more responsible for whole-body thermogenesis. No significant alterations in thermogenic genes were detected in BAT of both genotypes after NE injection (S4A Fig). Collectively, these results suggested that *Oct3* deficiency improved thermogenic capacity in WAT in the presence of adrenergic stimuli that resulted in an increase in core body temperature, O_2_ consumption, and whole-body energy expenditure.

### Ablation of *Oct3* enhanced catecholamine-stimulated lipolysis and β-adrenergic signaling in WAT

**Fig 3:**
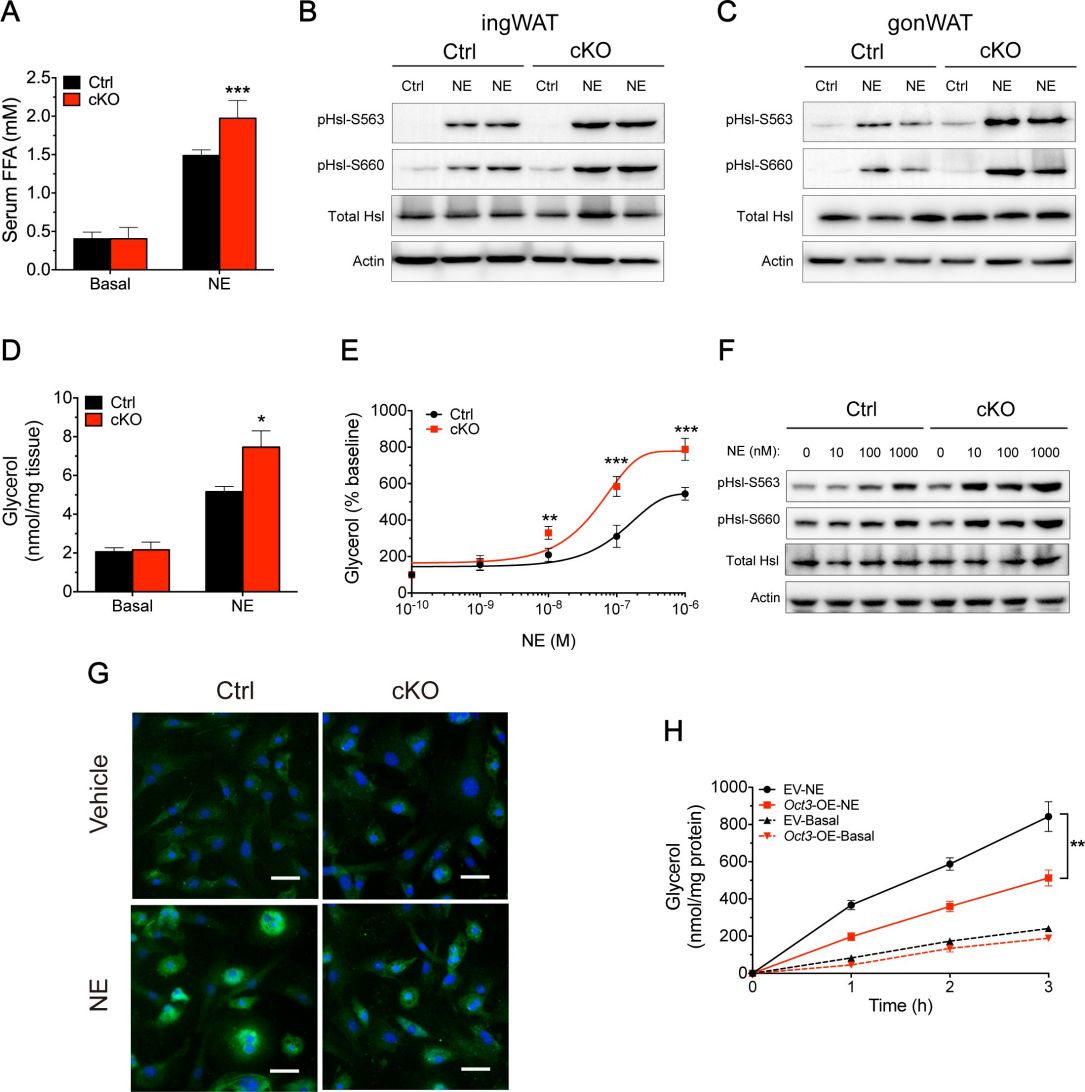
Genetic ablation of *Oct3* augmented NE-stimulated lipolysis in WAT. (A) Basal and NE-stimulated lipolysis by measuring serum FFAs in Ctrl and cKO mice (*n* = 6). (B, C) Protein levels of Hsl phosphorylated at Ser563 and Ser660 (pHsl-S563 and pHsl-S660, respectively) and total Hsl in ingWAT (panel B) and gonWAT (panel C) of Ctrl and cKO mice with NE (“NE”) or without NE injection (“Ctrl”). (D) Ex vivo glycerol release from AT explants in the absence (“basal”) or presence of NE (“NE”) (*n* = 4). (E) In vitro glycerol release from differentiated SVF-derived adipocytes from Ctrl and cKO mice in the absence or presence of NE (*n* = 4). (F) Protein levels of pHsl-S563, pHsl-S660, and total Hsl in differentiated SVF-derived adipocytes from Ctrl and cKO mice in the absence or presence of NE. (G) Immunofluorescence analysis of intracellular NE (green) in differentiated SVF-derived adipocytes from Ctrl and cKO mice. Adipocytes were either untreated or treated with NE (100 nM) for 10 minutes. DAPI (blue), nucleus. Scale bar, 50 μm. (H) Basal and NE-stimulated lipolysis, as measured by glycerol release from differentiated 3T3-L1 adipocytes stably transfected with EVs or *Oct3* (*Oct3-*OE) (*n* = 3). Data in panels A, D, E, and H were analyzed by Student *t* test. The numerical data underlying this figure are included in S1 Data. cKO, conditional knockout; Ctrl, control; EV, empty vector; FFA, free fatty acid; gonWAT, gonadal white adipose tissue; Hsl, hormone-sensitive lipase; ingWAT, inguinal white adipose tissue; KO, knockout; NE, norepinephrine; Oct3, organic cation transporter 3; OE, over-expression; pHsl, phosphorylated hormone-sensitive lipase; SVF, stromal vascular fraction; WAT, white adipose tissue.

Activation of β-AR signaling has been found to promote lipolysis via the PKA-mediated phosphorylation of Hsl. To investigate the role of Oct3 in catecholamine signaling in lipolysis, we tested in vivo lipolysis by measuring serum concentration of FFAs. There was no difference in basal lipolysis, while NE injection enhanced lipolysis more significantly in adipose-specific *Oct3* KO mice than in Ctrl (Fig 3A). Epinephrine had a similar effect on lipolysis in vivo (S4B Fig). Increased levels of Hsl phosphorylation at two PKA-responsive serine residues (pHsl-S563 and pHsl-S660) were observed in ingWAT (Fig 3B) and gonWAT (Fig 3C) of adipose-specific *Oct3* KO mice 4 hours after NE stimulation, which is necessary for Hsl translocation into lipid droplets and NE-induced lipolysis in rodents [24]. No noticeable difference was observed in Hsl phosphorylation 4 hours after injection in BAT between the two genotypes (S4C Fig). One possible explanation for these results was that deletion of *Oct3* led to impaired NE uptake into adipocytes and extracellular NE accumulation. Therefore, excess NE would activate β_3_-AR, increase cAMP concentration, and phosphorylate PKA, which would promote lipolytic activity via phosphorylating Hsl at S563 and S660 [25].

Next, we compared basal and NE-stimulated glycerol release from WAT explants derived from adipose-specific *Oct3* KO mice and Ctrl mice. The rates of glycerol release were similar under basal conditions in both genotypes, but were enhanced more in *Oct3* KO AT when treated with NE (Fig 3D). Ablation of *Oct3* in primary adipocytes led to an elevated response to adrenergic stimulation, as demonstrated by an increased lipolytic response to NE (Fig 3E) and intracellular signaling molecule activation (pHsl-S563 and pHsl-S660) (Fig 3F).

To compare NE uptake capacity in Ctrl and *Oct3 *KO adipocytes, we assayed intracellular NE levels using immunofluorescence. Primary adipocytes were treated with catecholamine-degradation enzyme inhibitors decynium-22 in the presence or absence of NE and were then incubated with anti-NE antibodies. Staining patterns were similar between unstimulated adipocytes; however, *Oct3* KO adipocytes had lower signals compared with Ctrl under NE stimulation, indicating that ablation of Oct3 in adipocytes impaired NE uptake (Fig 3G).

We next investigated the role of Oct3 on lipolysis in vitro by using a gain-of-function approach. Preadipocytes were transfected to produce *Oct3*-overexpressing 3T3-L1 cells (*Oct3*-OE). Although *Oct3* overexpression did not significantly affect adipocyte differentiation based on Oil Red O staining (S4D Fig) or measurement of basal lipolysis (Fig 3H), *Oct3*-OE cells displayed attenuated NE-stimulated lipolysis compared with EV cells (Fig 3H), likely a result of enhanced NE transport to adipocytes accompanied by reduction of extracellular NE. In addition, *Oct3*-OE and EV cells were treated with the adenylyl cyclase agonist forskolin (10 μM), and similar lipolytic capacities were observed (S4E Fig), indicating that cAMP signaling (blocked by forskolin) and downstream lipolytic capacity were not primary causes of the difference in NE-stimulated lipolysis in our model.

In light of the enhanced lipolysis in *Oct3* KO AT after NE stimulation, we examined the effect of adipose-specific KO of *Oct3* on the liver. Fatty liver was not observed (S4F Fig), and there was no significant difference in liver triglyceride levels between genotypes 4 hours after NE injection (S4G Fig). One possible reason was that single NE injection represented only a short-term effect on lipolysis and FFA flux in blood, and another was that the 4-hour duration time might be insufficient to generate significant differences in fat accumulation in the liver.

### *Oct3* deficiency induced white to brown fat transition and enhanced thermogenesis

**Fig 4:**
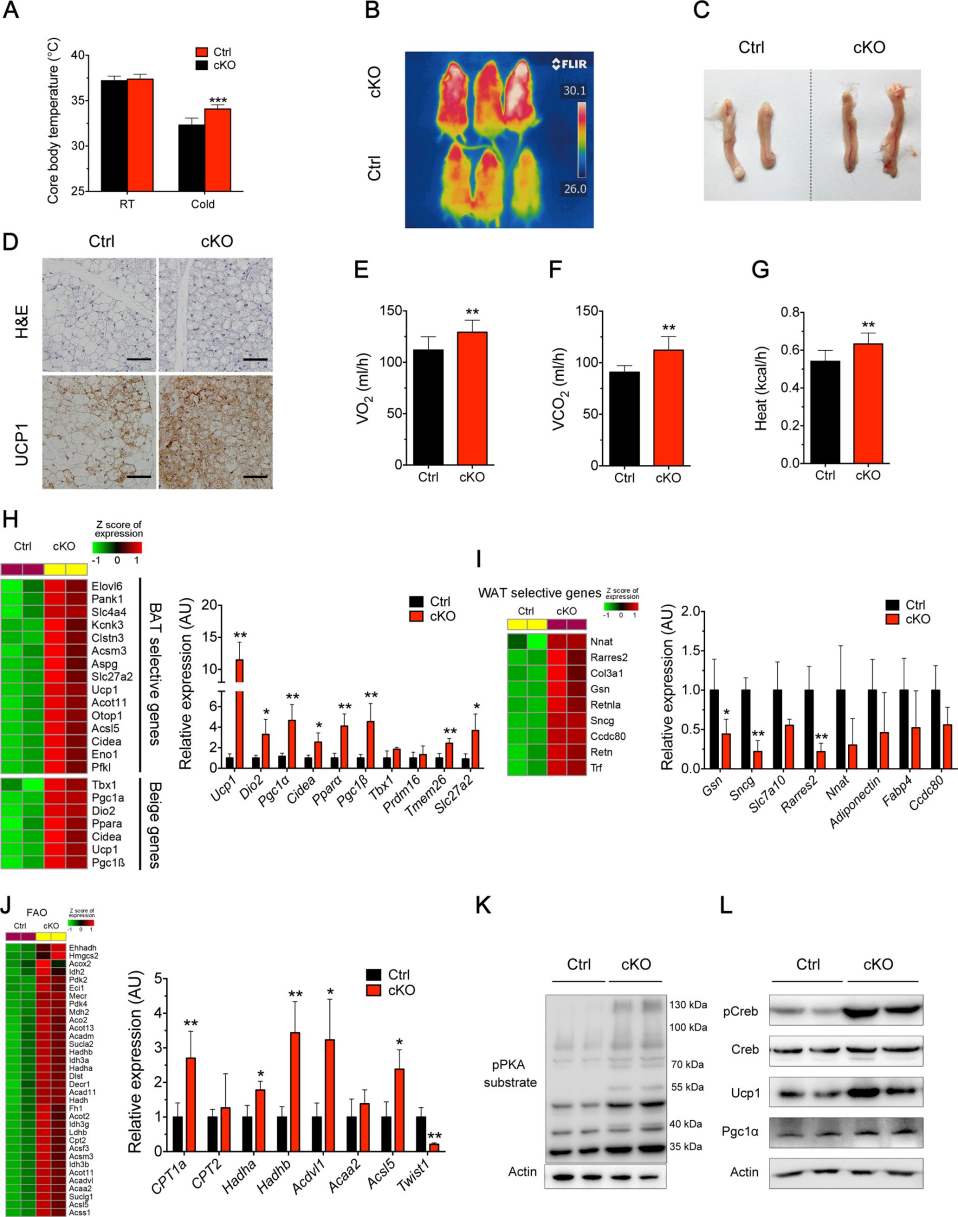
*Oct3* ablation promoted beiging and enhanced thermogenesis in ingWAT. Ctrl and cKO mice were housed at 4°C for 1 month. (A) Core body temperature of Ctrl and cKO mice at RT and after cold acclimation (*n* = 6). (B) Representative thermal images of Ctrl and cKO mice after cold acclimation. (C) Gross images of ingWAT of Ctrl and cKO mice. (D) Representative HE and Ucp1 immunohistochemical staining in ingWAT. Scale bar, 50 μm. Shown are representative images of three experiments. (E–G) Metabolic parameters of Ctrl and cKO mice after cold acclimation (*n* = 4). (E) O_2_ consumption; (F) CO_2_ production; (G) Heat production. (H–J) Heat maps (left) and real-time PCR analysis (right) of relative gene expression levels in ingWAT of Ctrl and cKO mice after cold acclimation (*n* = 6). Expression values in heat maps were z-transformed and scaled. (H) BAT selective genes and beige genes; (I) WAT selective genes; (J) Fatty acid β-oxidation genes. (K, L) Protein levels of pPKA substrate (panel K), pCreb, Creb, Ucp1, and Pgc1α (panel L) in ingWAT of Ctrl and cKO mice. Data in A and E–J were analyzed by Student *t* test. The numerical data underlying this figure are included in S1 Data. BAT, brown adipose tissue; cAMP, cyclic adenosine monophosphate; cKO, conditional knockout; Creb, cAMP-responsive element binding protein; Ctrl, control; HE, hematoxylin–eosin; ingWAT, inguinal white adipose tissue; Oct3, organic cation transporter 3; pCreb, phosphorylated cAMP-responsive element binding protein; pPKA, phosphorylated protein kinase A; RT, room temperature; Ucp1, uncoupling protein 1; VO_2_, oxygen consumption; WAT, white adipose tissue.

To study the role of *Oct3* in physiologically stimulated thermogenesis, adipose-specific *Oct3* KO and Ctrl mice were exposed to a 4°C ambient environment for 1 month. Both groups had similar rectal temperatures under RT (Fig 4A). However, adipose-specific *Oct3* KO mice showed significantly higher rectal temperatures (by 1.7°C) than those of Ctrl littermates after prolonged cold acclimation (Fig 4A and 4B). As seen in S5A Fig, body weight of adipose-specific *Oct3* KO mice had a slight but significant decrease compared with that of Ctrl mice after sustained cold exposure. We also observed a more reddish appearance of ingWAT in adipose-specific *Oct3* KO mice compared with Ctrl (Fig 4C). Notably, adipose-specific *Oct3* KO ingWAT contained more clusters of uncoupling protein 1 (Ucp1)-positive multilocular adipocytes than Ctrl after cold exposure (Fig 4D). Whole-body energy expenditure of mice after cold challenge was measured by Comprehensive Lab Animal Monitoring System (CLAMS). Adipose-specific *Oct3* KO mice showed markedly higher O_2_ consumption rates (OCRs) (Fig 4E), CO_2_ production rates (Fig 4F), and heat production (Fig 4G) than Ctrl, indicating a higher BMR. In addition, higher NE levels were observed in *Oct3* KO ingWAT (S5B Fig), probably due to accumulation of extracellular NE derived from sympathetic nerve endings during cold challenge. Meanwhile, NE concentration in serum was also slightly increased, probably due to a leakage of extracellular NE in WAT into blood circulation (S5C Fig). These results showed the role of Oct3 in altering NE distribution between adipocytes and blood circulation.

In order to examine global gene expression changes in ingWAT, RNA from ingWAT of both genotypes were subjected to RNA sequencing (RNA-seq) analysis. Ablation of *Oct3* led to significantly differential expression of 2,521 genes in ingWAT (adjusted *p* < 0.05) (S5D Fig and S1 Table). Widespread up-regulation of BAT-selective and beige genes were identified in *Oct3* KO ingWAT (Fig 4H), while WAT-selective genes showed a down-regulation tendency (Fig 4I). Furthermore, expression of genes involved in fatty acid oxidation (FAO) also showed substantial up-regulation (Fig 4J). Based on the RNA-seq data analysis, we conducted real-time PCR analysis on genes involved in thermogenesis and adipose physiology. *Oct3* KO ingWAT from cold-exposed mice had significantly higher mRNA expression levels of thermogenic genes *Ucp1*, *Dio2*, *Pgc1α*, *Cidea*, *Pparα*, *Pgc1β*, *Tmem26*, and *Slc27a2*—as well as genes that showed an up-regulation trend, including *Tbx1* (Fig 4H)*—*whereas WAT-selective genes *Gsn*, *Sncg*, *and Rarres2* were significantly decreased (Fig 4I). Several key genes of FAO were significantly up-regulated in *Oct3* KO ingWAT, including *Cpt1a*, *Hadha*, *Hadhb*, *Acdvl1*, and *Acsl5* (Fig 4J). Moreover, a negative regulator of FAO, *Twist1*, was significantly reduced in *Oct3* KO ingWAT, by 80% (Fig 4J). No significant changes in morphology of BAT and gonWAT from both genotypes were observed (S5E Fig), consistent with unchanged mRNA levels of *Pgc1α*, *Dio2*, and other thermogenesis genes, as well as representative FAO genes (S5F and S5G Fig).

Given the close relationships between Oct3, NE, and thermogenesis, we next focused on the NE/β-AR/cAMP pathway. Western blot analysis showed that phospho-PKA (pPKA) substrate (Fig 4K), phospho-Creb (pCreb), and downstream Ucp1 and Pgc1α levels (Fig 4L) were significantly elevated in ingWAT from adipose-specific *Oct3* KO mice compared with Ctrl. Protein levels of pCreb, Ucp1, and Pgc1α remained unchanged in BAT (S5H Fig) and gonWAT (S5I Fig). Moreover, genes encoding components of the cAMP/PKA pathway (*Adcy3* and *Adcy10*) were also up-regulated in ingWAT after cold exposure (S5J Fig). Based on the data above, we hypothesized that the enhanced browning effect in adipose-specific *Oct3* KO mice during cold challenge may be due to higher metabolic rate and elevated thermogenic gene expression.

### Ablation of *Oct3* promoted mitochondria biogenesis and a remodeling of energetic metabolism in ingWAT during cold challenge

**Fig 5:**
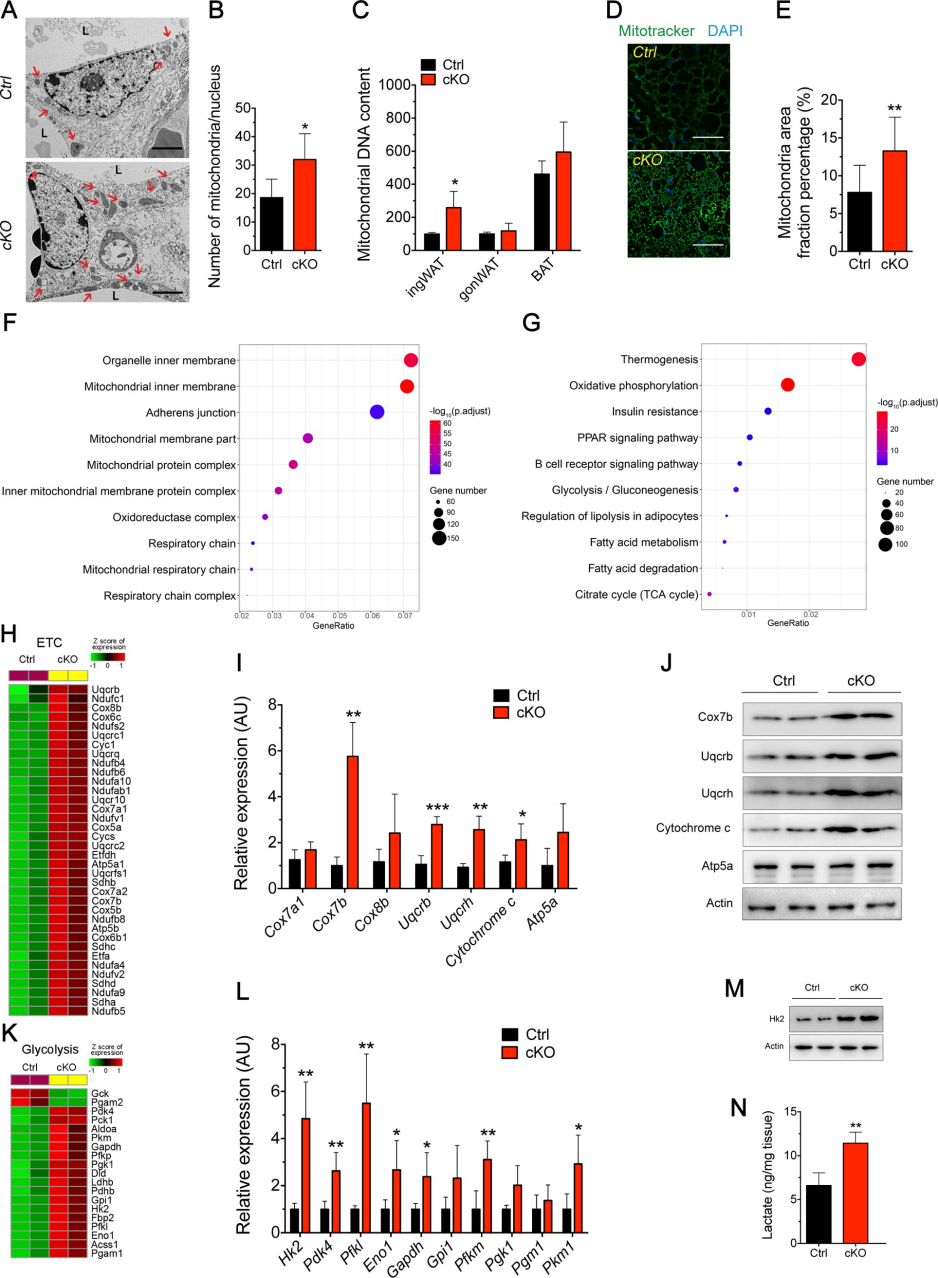
Deletion of *Oct3* induced mitochondria biogenesis and enhanced mitochondria function and glycolysis in ingWAT. Ctrl and cKO mice were housed at 4°C for 1 month. (A) TEM images of ingWAT sections. Red arrows indicate mitochondria. (B) Mitochondrial number per nucleus determined from electron micrographs. (C) Mitochondrial DNA content determined in ingWAT, gonWAT, and BAT (*n* = 4). (D) Representative figures of MitoTracker staining in ingWAT. (E) Positive signals of MitoTracker quantified as percentage total cells. (F, G) ClusterProfiler package in R was used to test for enrichment of cellular component (panel F) and KEGG pathway (panel G). Dot color indicates statistical significance (−log_10_p.adjust value); dot size represents the gene numbers annotated to each term. (H) Heat maps of relative expression levels of ETC genes in ingWAT of Ctrl and cKO mice. (I) mRNA expression of genes associated with mitochondrial respiratory chain complex genes and other mitochondrial genes in ingWAT (*n* = 6). (J) Western blots of mitochondrial respiratory chain complex proteins in ingWAT. (K) Heat maps of relative expression levels of glycolytic genes in ingWAT of Ctrl and cKO mice. (L) mRNA expression of genes associated with glycolysis in ingWAT (*n* = 6). (M) Western blots of proteins associated with glycolysis in ingWAT. (N) The amount of lactate in ingWAT. Data in B–C, E, I, L, and N were analyzed by Student *t* test. All images shown are representative of three experiments. The numerical data underlying this figure are included in S1 Data. AU, arbitrary unit; BAT, brown adipose tissue; cKO, conditional knockout; Ctrl, control; ETC, electron transport chain; gonWAT, gonadal white adipose tissue; ingWAT, inguinal white adipose tissue; KEGG, Kyoto Encyclopedia of Genes and Genomes; Oct3, organic cation transporter 3; TEM, transmission electron microscope.

We next characterized the effect of *Oct3* deletion on mitochondria in ingWAT after cold exposure. Electron microscopy showed more accumulated mitochondria and enlarged mitochondrial area (Fig 5A), a larger number of mitochondria per cell area (Fig 5B), and increased mitochondrial DNA content (Fig 5C) in *Oct3* KO adipocytes from ingWAT compared with Ctrl. Larger surface area of mitochondria was also observed in *Oct3* KO ingWAT stained with Mitotracker (Fig 5D and 5E). Consistently, the gene ontology (GO) analysis of RNA-seq data revealed that differential genes associated with mitochondrial component were enriched (Fig 5F and S1 Table). Moreover, Kyoto Encyclopedia of Genes and Genomes (KEGG) pathway analysis highlighted specific changes in genes associated with oxidative phosphorylation, glycolysis and fatty acid metabolism, and citric acid cycle (Fig 5G and S1 Table).

In ingWAT of adipose-specific *Oct3* KO mice, the increase in cold-induced thermogenesis was coupled with broad up-regulation of electron transport chain (ETC) genes (Fig 5H). Real-time PCR analysis showed that expression of mitochondrial respiratory chain complexes genes (*Cox7b*, *Uqcrb*, *Uqcrh*, and *Cytochrome c*) were remarkably increased in *Oct3* KO ingWAT compared with Ctrl under cold stimulation (Fig 5I). In line with these changes, western blot analysis showed that protein levels of cytochrome c oxidase subunit 7b (Cox7b), Uqcrb, Uqcrh, and Cytochrome c were significantly elevated in *Oct3* KO ingWAT from mice under cold exposure (Fig 5J). We found no difference in the expression of ETC genes in BAT (S5K Fig) and gonWAT (S5L Fig) between genotypes.

The profound differences of glycolytic genes in *Oct3* KO ingWAT suggested that *Oct3* deletion resulted in a metabolic rewiring during cold exposure (Fig 5K). Real-time PCR analysis of ingWAT showed up-regulation of glycolytic genes (*Hk2*, *Pdk4*, *Pfkl*, *Eno1*, *Gapdh*, *Pfkm*, and *Pkm1*) in adipose-specific *Oct3* KO versus Ctrl mice (Fig 5L). The protein levels of a key enzyme of glycolysis, Hk2, and the amount of lactate (the product of glycolysis) were up-regulated in ingWAT from adipose-specific *Oct3* KO mice (Fig 5M and 5N). These alterations were selective in ingWAT, as no significant difference between groups was observed in BAT (S5K and S5M Fig) or gonWAT (S5L and S5M Fig).

Collectively, our results suggested that the elevated browning effect in adipose-specific *Oct3* KO mice may be owing to increased energy expenditure, mitochondrial biogenesis, and enhanced glycolysis in ingWAT.

### Enhanced thermogenesis and lipolysis resulted from overactivating the NE/β-AR/PKA signaling pathway in adipose-specific *Oct3* KO mice

**Fig 6:**
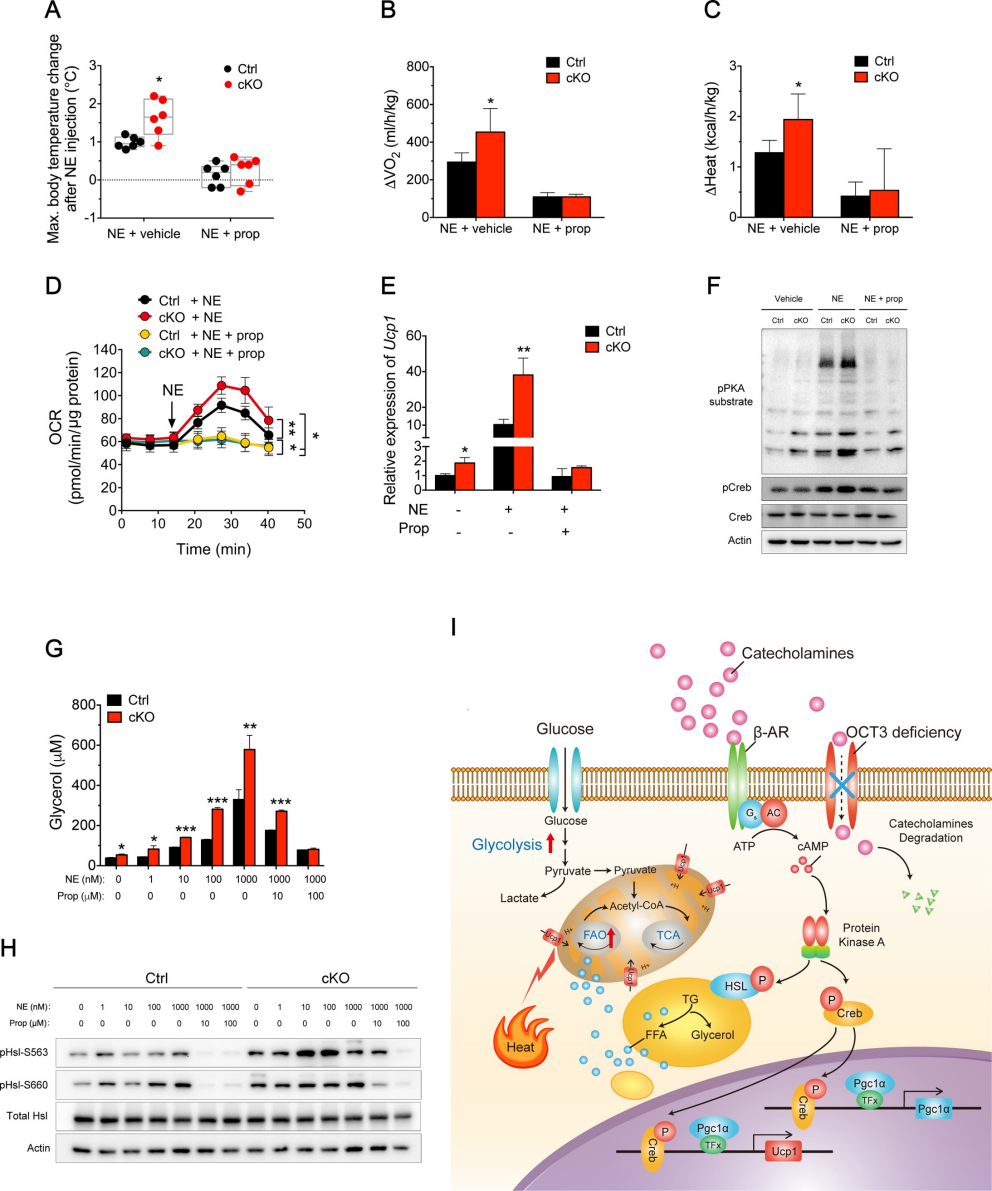
*Oct3* deficiency induced a thermogenesis and lipolysis program through the NE/β-AR/PKA signaling pathway. (A–C) Maximal body temperature change (panel A), maximal O_2_ consumption change (ΔO_2_) (panel B), and maximal heat production change (ΔHeat) (panel C) of Ctrl and cKO mice (*n* = 6 in body temperature measurement; *n* = 4 for oxygen consumption and heat production measurement). NE was injected into mice with propranolol (“NE + prop”) or without propranolol (5 mg/kg, s.c.) pretreatment (“NE + vehicle”). (D) The OCR in *Oct3* KO beige adipocytes and Ctrl. Cells were acutely stimulated with NE in the absence or presence of propranolol at the indicated time point (arrow). Ctrl or *Oct3* KO adipocytes with NE (*n* = 7 for both); Ctrl or *Oct3* KO adipocytes with NE and propranolol (*n* = 8 for both). (E) *Ucp1* mRNA expression in differentiated primary inguinal adipocytes from Ctrl and cKO mice after stimulation with NE and T_3_ in the presence or absence of propranolol (*n* = 3). (F) Western blotting of pPKA substrate, pCreb, and total Creb in primary inguinal adipocytes from Ctrl and cKO mice stimulated with NE in the presence or absence of propranolol. (G) In vitro glycerol release from differentiated SVF-derived adipocytes in the presence or absence of NE and propranolol (*n* = 3). (H) Western blotting of pHsl-S563, pHsl-S660, and total Hsl in differentiated SVF-derived adipocytes from Ctrl and cKO mice in the presence or absence of NE and propranolol. (I) Schematic diagram of Oct3-regulating thermogenesis and lipolysis in ingWAT. Data in A–E and G were analyzed by one-way ANOVA followed by Tukey’s test. Data in I and K were analyzed by Student *t* test. The numerical data underlying this figure are included in S1 Data. β-AR, β-adrenergic receptor; AC, adenylyl cyclase; cAMP, cyclic adenosine monophosphate; Creb, cAMP-responsive element binding protein; Ctrl, control; FFA, free fatty acid; G_s_, G_s_ alpha subunit; Hsl, hormone-sensitive lipase; ingWAT, inguinal white adipose tissue; KO, knockout; NE, norepinephrine; OCR, O_2_ consumption rate; Oct3, organic cation transporter 3; pCreb, phosphorylated cAMP-responsive element binding protein; pHsl, phosphorylated hormone-sensitive lipase; PKA, protein kinase A; pPKA, phosph-PKA; prop, propranolol; s.c., subcutaneous; SVF, stromal vascular fraction; T_3_, triiodothyronine; TF, transcription factor; TG, triglyceride; VO_2_, oxygen consumption.

To further investigate the relationship between *Oct3*-depletion–induced NE-stimulated thermogenesis and β-AR signaling, we firstly cotreated mice with NE and propranolol, a pharmacological β-AR inhibitor. An elevated thermogenic response in adipose-specific *Oct3* KO mice was observed, including a more significant increase in core body temperature (Fig 6A), O_2_ consumption (Fig 6B), and heat production (Fig 6C) following single NE treatment, as described before (Fig 2A–2H). However, propranolol pretreatment abolished the elevation of thermogenic response (Fig 6A–6C), indicating that the increased energy expenditure in adipose-specific *Oct3* KO mice was mediated by the β-AR signaling pathway.

To estimate the functional relevance of Oct3 for thermogenic capacity of adipocytes, we performed plate-based respirometry on primary adipocytes from preadipocytes isolated from SVC of ingWAT, which maintained expression of beige-related genes. Although the OCR curves were nearly identical in primary adipocytes of both genotypes in the absence of NE (S6A Fig), *Oct3* KO beige adipocytes displayed a higher OCR than that of Ctrl cells after NE stimulation (Fig 6D). Additionally, pharmacological inhibition of β-AR by propranolol significantly blunted the NE-stimulated OCR increase to a similar extent in both groups (Fig 6D).

Primary adipocytes derived from ingWAT were treated with NE and triiodothyronine (T_3_) to induce *Ucp1* expression [26]. An approximate 1.9-fold increase in *Ucp1* mRNA expression in *Oct3* KO beige adipocytes was found even in the absence of NE compared with Ctrl, and another 3.7-fold increase occurred in response to NE treatment (Fig 6E). Meanwhile, acute propranolol-induced inhibition of β-AR similarly blunted the NE-induced increase in *Ucp1* transcripts in the beige both groups of adipocytes (Fig 6E). As a control, no noticeable difference was observed in mRNA expression of the NE-insensitive gene *Ap2* between groups (S6B Fig). Additionally, *Ucp1* transcripts and Creb phosphorylation showed no response to treatment of other Oct3 substrates (epinephrine, dopamine, and serotonin) (S6C and S6D Fig).

As NE was known to activate β-AR and trigger PKA-Creb signaling, we examined whether the enhanced thermogenesis in *Oct3* KO beige adipocytes was regulated by the PKA/Creb pathway. NE-stimulated *Oct3* KO adipocytes showed robust phosphorylation of PKA substrates and Creb, without changes in total Creb (Fig 6F). Meanwhile, these effects were largely eliminated by blockade of β-AR signaling (Fig 6F), indicating that β-AR signaling was required for enhanced thermogenic activity under *Oct3* deficiency.

We next aimed to determine the requirement of NE/β-AR pathway in lipolysis induced by *Oct3* ablation. Propranolol at 10 μM partially but significantly inhibited the enhancing effect of NE on lipolysis and Hsl phosphorylation, while 100 μM propranolol showed complete inhibition in *Oct3* KO adipocytes (Fig 6G and 6H), indicating that this effect relied on β-AR function.

Based on these collective results, we propose the following model (Fig 6I). Oct3 locates to cell membranes of adipocytes and uptakes NE, which thereby decreases NE concentration in AT microenvironments. *Oct3* deficiency reduces clearance of extracellular NE, subsequently increasing extracellular NE concentration and leading to enhanced lipolysis, glycolysis, thermogenesis, and WAT browning via β-AR/cAMP/PKA pathway activation.

### Adipose-specific *Oct3* KO mice fed with high-fat diet showed enhanced thermogenesis, lipolysis, and improved insulin sensitivity under cold exposure

Next, we explored the effect of adipose-specific *Oct3* KO on diet-induced obesity (DIO). There was no difference in body weight between adipose-specific *Oct3* KO and littermate Ctrl mice fed with a high-fat diet (HFD) and housed at thermoneutrality (30°C) (S7A Fig) or at RT (S7B Fig) for 12 weeks. We then subjected HFD-fed mice of both genotypes (housed at thermoneutrality for 12 weeks) to 4°C cold exposure. Higher body temperature was evident in adipose-specific *Oct3* KO mice after the first 4 hours (S7C Fig). After 1-week cold acclimation, the body weights of adipose-specific *Oct3* KO mice decreased more (S7D Fig) despite no difference in food intake (S7E Fig), serum glucose (S7F Fig), triglyceride (S7G Fig), cholesterol (S7H Fig), insulin (S7I Fig), and adiponectin (S7J Fig). Meanwhile, serum FFAs increased about 21% during cold stimulation in adipose-specific *Oct3* KO mice (S7K Fig), implying higher lipolysis owing to lower NE clearance under Oct3 deficiency. Furthermore, glucose tolerance was improved in adipose-specific *Oct3* KO mice compared with Ctrl mice (S7L Fig). These data showed that thermogenesis and lipolysis under cold stimuli were still enhanced in adipose-specific *Oct3* KO mice treated with HFD, and insulin sensitivity was also improved.

### Human *OCT3* polymorphisms were associated with BMR

To determine the relevance of the studies in mice to human, BMR with polymorphisms within the human *OCT3* locus was explored through mining of GWAS Catalog, dbGAP, and UK BioBank databases. Among the 11 single-nucleotide polymorphisms (SNPs) in the *OCT3* locus, four independent SNPs were associated with BMR with *p* < 5 × 10^−8^ (S8A Fig and S3 Table). From the GTEx Portal, we noted that 10 of the 11 SNPs are significantly associated with human *OCT3* expression levels in ATs (subcutaneous and/or visceral omentum) (S8B and S8C Fig). Each of the alleles, which was associated with lower expression levels of *OCT3* in AT, was correlated with higher BMR in the UK Biobank participants.

To further confirm the relevance to humans of the studies in mice, we performed experiments on human AT and primary human AT-derived mesenchymal stem cells (ATMSCs) that were induced to differentiate into adipocytes. Both human AT and differentiated ATMSCs showed high expression levels of OCT3 and NE-degrading enzymes (S8D–S8F Fig). These results were consistent with studies on human adipocytes showing that NE uptake existed and was inhibited by OCT3 inhibitors [20]. Furthermore, inhibition of OCT3 led to an increased lipolytic response to NE in differentiated ATMSCs (S8G Fig), similar to the observations in mouse stromal vascular fraction (SVF)-derived adipocyte model (Fig 4E).

Although human functional genomic data and cell studies provided cues for metabolic roles of OCT3, further investigations are needed to define the subtle roles of human OCT3 in AT.

## Discussion

Brown and beige AT are two key drivers for the dissipation of chemical energy to stimulate thermogenesis [27]. The current study demonstrates that genetic deletion of *Oct3* enhances ingWAT beiging and up-regulates NE-induced thermogenesis, lipolysis, and mitochondria biogenesis by attenuating NE clearance. These findings support the non-neural role of Oct3 as a catecholamine scavenger for beige AT microenvironments in adaptive thermogenesis.

Recent studies have proposed a neural mechanism for NE uptake by Slc6a2 in special macrophages in AT [14, 15]. However, a proinflammatory state seems necessary to enhance their NE uptake in macrophages [14]. In addition, considering the high-affinity, low-capacity nature of Slc6a2 (K_m_: 0.28 ± 0.03 μM; V_max_: 5.83 ± 0.49 pmol/mg protein/min) [23], a NE transport system with greater transport capacity may be needed to meet increased metabolic demand during long-term cold challenge. With high expression in adipocytes and high capacity for catecholamines, Oct3 (K_m_: 0.183 ± 0.0275 mM; V_max_: 3.57 ± 0.174 nmol/mg protein/min) may function in part to clear catecholamines in white AT microenvironments. However, the quantitative contribution and exact conditions of extraneuronal NE uptake through Oct3 versus neuronal NE clearance remains to be investigated.

Cold challenge induces stronger browning and NE/β-AR/PKA signaling in ingWAT of adipose-specific *Oct3* KO mice. Consistent with sustained action of catecholamines, protein levels of pPKA substrate, pCreb, and downstream *Ucp1* and *Pgc1α* are all up-regulated. Pgc1α is the crucial transcriptional co-activator that is highly induced after cold exposure [28]. In white adipocytes, ectopic expression of Pgc1α induces *Ucp1* expression and essential enzymes in mitochondria ETC and also increases mitochondrial DNA content [28, 29]. Mitochondria biogenesis and expression of ETC is significantly increased in ingWAT of adipose-specific *Oct3* KO mice after cold challenge, which may result from elevated Pgc1α protein expression. Moreover, genes encoding fatty acid β-oxidation were exclusively up-regulated in ingWAT of *Oct3*-deficient mice after cold exposure. Consequently, more FFAs may be utilized as the primary fuel source for thermogenesis through Ucp1. These chain reactions are likely driven by more NE available due to *Oct3* deletion.

During browning of ingWAT in adipose-specific *Oct3* KO mice, metabolic rewiring of glucose metabolism to glycolysis is another significant phenomenon observed. Upon cold exposure, key enzymes in glycolysis and pyruvate dehydrogenase kinase 4 (*Pdk4*, the key regulatory enzyme linking glycolysis to the citric acid cycle) are significantly up-regulated in ingWAT of adipose-specific *Oct3* KO mice. Higher expression of *Pdk4* restricts the conversion of glucose to acetyl-CoA and reprograms it towards a higher glycolytic metabolism [30]. Along with other key glycolytic enzymes, the enhanced glycolysis in adipose-specific *Oct3* KO mice may compensate for the loss of mitochondrial ATP production due to heat-generating mitochondrial uncoupling [31]. Recent reports have demonstrated that expression of glycolytic genes and *Pdk4* are induced by β-AR agonists in BAT [32]. The enhanced glycolytic activity in ingWAT of cold-exposed adipose-specific *Oct3* KO mice most likely results from stronger adrenergic stimulation through blunted NE catabolism by Oct3 deficiency. To date, there is little information available regarding the role of glucose metabolism in WAT. RNA-seq data also showed that these glycolytic genes are more highly expressed in ingWAT in adipose-specific *Oct3* KO mice after cold exposure (S1 Table). These findings revealed that Oct3-mediated glucose metabolism is important to WAT energy homeostasis.

The differential responsiveness of WAT and BAT in adipose-specific *Oct3* KO mice may result from several factors. First, *Oct3* has a much lower expression in BAT compared with WAT. Second, current studies suggest that BAT is more densely innervated compared with WAT [33], and neuronal *Slc6a2* may be largely responsible for the clearance of catecholamines released in BAT after NE injection or cold exposure [34]. Although browning of WAT can lead to metabolic improvements, the marked increase in energy expenditure (Fig 4E–4G) may also come from other sources such as skeletal muscle, cardiac and respiratory work, and liver, stimulated by enhanced circulating NE level [30–32]. Under HFD treatment at RT or thermoneutrality, similar body weights between two genotypes are observed. It is probably due to drastically reduced density of sympathetic nerves in ingWAT under HFD [35]. On the other hand, the Uptake_1_ system might be sufficient to compensate for the KO effect of *Oct3*. The overall proposed mechanism for Oct3 effects on WAT browning is summarized in Fig 6I.

Exploration of the Gene Atlas database for genetic associations of human BMR with variants in *OCT3* shows consistency with some of the results in the adipose-specific *Oct3* KO mouse model. In particular, several reduced expression variants of OCT3 are positively correlated with BMR in human. Though a greater baseline BMR is not observed in the adipose-specific *Oct3* KO mice, metabolic rate is greater after either NE administration or cold challenge. Our study suggests an important clinical implication as β_3_-AR plays a central role in regulating nonshivering thermogenesis, which enhances energy expenditure and has potential to treat obesity [36]. Inhibition of OCT3 might block NE transport into adipocytes and mimic β_3_-AR agonists, resulting in browning of WAT and enhanced lipolysis. In summary, our results demonstrate that Oct3 is essential in catecholamine clearance in adipocytes of WAT and blunts intracellular responses upon sustained catecholamine stimulation by regulating the activity of β-AR. Development of OCT3-specific inhibitors may shed light on improving metabolic disorders.

## Materials and methods

### Ethics statement

All experiments were performed in accordance with guidelines of the Institute for Laboratory Animal Research of Tsinghua University. The experimental procedures were approved by the Administrative Committee of Experimental Animal Care and Use of Tsinghua University, licensed by the Science and Technology Commission of Beijing Municipality (SYXK-2014-0024), and they conformed to the National Institutes of Health guidelines on the ethical use of animals.

### Animals

Adiponectin-Cre mice were obtained from the Jackson Laboratory (stock NO.010803). *Oct3*^*fl/fl*^ mice were generated in the Ying Xu lab (Soochow University), backcrossed with C57BL/6J mice for at least 8 generations, and subsequently intercrossed with Adiponectin-Cre mice. Mice were housed under a 12-hour light/dark cycle (light period 7:00–19:00) at constant temperature (22°C RT). Food and water were available ad libitum. Male mice were 8 to 10 weeks of age when used for experiments.

### Uptake assays

HEK-293 Flp-In cells (Invitrogen, Carlsbad, CA) stably overexpressing human or mouse *OCT3* were generated, maintained, and used for uptake assays as previously described [37]. For comparison between human OCT3 and mouse Oct3 NE uptake kinetics (Fig 2E), uptake time was 2 minutes at 37°C. *K*_*m*_ and *V*_*max*_ were calculated by fitting the data to Michaelis-Menten equations using GraphPad Prism software (La Jolla, CA). For [^3^H]-NE uptake in primary adipocytes, the same procedures were performed as described above.

### Cold challenge and indirect calorimetry

For cold challenge experiments, male mice were placed at 16°C for 1 week, and then 3 weeks at 4°C. Metabolic measurements were measured using CLAMS (Columbus Instruments, Columbus, OH). Mice were individually housed in cages. Food and water were provided ad libitum.

### Mouse Oct3 three-dimensional–structure model and catecholamine docking

The amino acid sequence of the mouse Oct3 was retrieved from the UNIPROT database (Q9WTW5) [38]. Homology models of Oct3 was generated via four steps. First, the primary sequence of Oct3 was submitted to the RCSB Protein Data Bank [39] to search for suitable template structures using PSI-BLAST [40]. Human glucose transporter 3 (hGLUT3) having its crystal structure determined in complex with D-glucose at 1.5 Å resolution in an outward-occluded conformation (PDB ID: 4ZW9) [41] is the closest structurally characterized protein to Oct3, sharing 24% sequence identity. Second, a sequence alignment between Oct3 and hGLUT3 was computed by Multiple Sequence Comparison by Log-Expectation (MUSCLE) [42]. Third, a total of 500 homology models were generated for Oct3 with the standard “automodel” class in MODELLER [43] on the basis of hGLUT3 template. Finally, these 500 models were evaluated by the discrete optimized protein energy (DOPE) score [44], and the best ranking model was selected.

Molecular docking screens against the selected Oct3 homology model was performed with a semi-automatic docking procedure. All docking calculations were performed with DOCK 3.6 [45]. The docking database contains seven compounds, dextroamphetamine, NE, epinephrine, dopamine, serotonin, histamine, and tyramine. The docked compounds were ranked by the docking energy that is the sum of van der Waals, Poisson-Boltzmann electrostatic, and ligand desolvation penalty terms.

### Histology and immunofluorescence analysis

Fresh tissue samples were fixed in 4% paraformaldehyde, dehydrated in serial alcohol, embedded in paraffin, cut into 5-μm–thick sections, and stained with hematoxylin–eosin (HE). Ucp1 immunohistochemical staining was performed as previously described [46]. Specimens were prepared and observed by transmission electron microscope (TEM) (H-7650; Hitachi, Japan) as previously described [47]. Mitochondria numbers were counted in more than 10 images for each specimen.

Mouse ingWAT frozen sections were then blocked for 30 minutes in PBS containing 10% normal goat serum at RT. For Oct3 staining, sections were incubated with a rabbit anti-mouse Oct antibody (1:200; OriGene Technologies, Rockville, MD) followed by staining with a Goat anti-Rabbit IgG (H+L) Cross-Adsorbed Secondary Antibody, Alexa Fluor 488 (1:500; Invitrogen, Carlsbad, CA). Then, sections were stained with DAPI for 10 minutes. Images were obtained with a Nikon A1 confocal microscope (Nikon Corp., Japan).

For mitochondria staining, deparaffinized slices of ingWAT from Ctrl and cKO mice were incubated with 250 nM MitoTracker (Molecular Probes, OR, USA) for 1 hour at RT. Slides were washed with PBS and had coverslips mounted. Images were obtained with a Nikon A1 confocal microscope (Nikon Corp., Japan). Mitochondria area fraction percentage was calculated as previously described [48] using NIS-Elements software (Nikon Corp., Japan). More than 20 fields were scored per group.

For detection of intracellular NE by immunofluorescence, primary adipocytes were incubated with medium containing NE (100 ng/ml) for 20 minutes at 37°C. After blocking, the adipocytes were next incubated with anti-noradrenaline (ab8887, 1:50, Abcam, Cambridge, MA) overnight at 4°C. The following procedures of immunofluorescence were described earlier.

### Differentiation of primary adipocytes and 3T3-L1 cells

Isolation of stromal-vascular cells from ingWAT and BAT was performed as described previously [49, 50]. Cells were cultured in growth medium DMEM/F-12 containing 10% FBS and 1% penicillin/streptomycin at 37°C with 5% CO_2_. For induction to differentiate, cells were incubated with induction medium containing 5 mg/ml insulin, 1 mΜ dexamethasone, 1 μM rosiglitazone, 0.5 mM 3-isobutyl-1-methylxanthine, 1 nM T_3_, and 125 nM indomethacin for 2 days. Cells were then maintained with 1 μM rosiglitazone and 1 nM T_3_ until maturation. Where indicated, cells were treated with T_3_ (10 μM), norepinephrine (100 nM) for 4 hours to induce *Ucp1* expression.

3T3-L1 preadipocytes were incubated with an induction medium DMEM containing 1 μg/ml insulin, 0.25 μM dexamethasone, 0.5 mM 3-isobutyl-1-methylxanthine, and 2 μM rosiglitazone for 2 days, and then with a differentiation medium DMEM containing 1 μg/ml insulin for another 2 days. Fresh DMEM was supplied every 2 days until maturation. For Oil Red staining, fully differentiated 3T3-L1 cells were fixed with 4% paraformaldehyde, followed by Oil Red O incubation for 2 hours.

### Determination of NE-induced thermogenesis

Ctrl and cKO mice (males, 8 weeks, chow diet) were intraperitoneally injected with pentobarbital (75 mg/kg), which does not inhibit nonshivering thermogenesis [51, 52]. For NE treatment, mice were subcutaneously injected with 0.3 mg/kg NE under anesthesia. For β-AR inhibition assay, propranolol (5 mg/kg) was given 20 minutes before NE. The core body temperature was recorded with a rectal probe connected to a digital thermometer (Yellow Spring Instruments). Infrared thermal images were obtained using a FLIR E60 compact infrared thermal imaging camera and were analyzed using FLIR Tools software (FLIR, North Billerica, MA).

### Determination of NE-induced lipolysis

For in vivo lipolysis, 0.3 mg/kg NE or saline was injected intraperitoneally into Ctrl and cKO mice. Tail blood was taken from mice 20 minutes after injection of NE or saline. Serum was separated for the determination of FFA using NEFA C kits (Wako, Oxoid SA, France).

For ex vivo lipolysis, about 50 mg WAT samples from overnight fasted mice were incubated in KRB buffer (12 mM HEPES, 121 mM NaCl, 4.9 mM KCl, 1.2 mM MgSO_4_, and 0.33 mM CaCl_2_) containing 2% FA-free BSA and 2.5 mM glucose, and then stimulated with 1 μM NE. Glycerol release was measured by using free glycerol reagent (Sigma-Aldrich, USA).

For in vitro lipolysis, glycerol release from differentiated SVF-derived or 3T3-L1 adipocytes was measured as previously described [53]. Lipolysis was stimulated by incubation of NE (1 μM, Sigma-Aldrich, USA) or forskolin (10 μM, Sigma-Aldrich, USA).

### In vivo uptake

Chemical sympathectomy was performed by unilateral denervation in ingWAT on one side and sham-operation on the collateral side to eliminate SNS NE release and uptake, as described previously [54]. The effectiveness of sympathectomy was confirmed by immunofluorescence of tyrosine hydroxylse (Th).

After 3 days of recovery from surgery, mice were given intraperitoneal injection of 0.5 mg/kg of NE spiked with [^3^H]-NE (PerkinElmer, CT, USA). After 30 minutes, mice were transcardially perfused with ice-cold PBS and washed for 4 to 5 minutes. IngWAT was immediately isolated with the major blood vessels removed and homogenized in Solvable (PerkinElmer CT, USA) overnight. Total radioactivity in ingWAT homogenates was determined by liquid scintillation counting. Protein content of homogenates was measured by BCA assay (Thermo Scientific, USA).

### RNA-seq analysis

For RNA-seq analysis of ingWAT, total RNA was extracted using the RNeasy Lipid Mini Kit (Qiagen, Hilden, Germany) based on the manufacturer’s instructions. Quality of RNA was analyzed using the Agilent 2100 (Agilent Technologies, Palo Alto, CA). The poly-A-containing mRNA was purified by using poly-T oligo-attached magnetic beads. Purified mRNA was fragmented into small pieces using divalent cations under elevated temperature. RNA fragments were copied into first-strand cDNA using reverse transcriptase and random primers, followed by second-strand cDNA synthesis using DNA polymerase I and RNase H. A single “A” base was added to cDNA fragments, and the adapter was subsequently ligated. The products were then purified and enriched with PCR amplification. PCR yield was quantified by Qubit, and samples were pooled together to make a single-strand DNA circle (ssDNA circle), which gave the final library.

DNA nanoballs (DNBs) were generated with the ssDNA circle by rolling circle replication (RCR) to enlarge the fluorescent signals at the sequencing process. The DNBs were loaded into the patterned nanoarrays and had 50 bp of single end read through the BGISEQ-500 platform. The DNB-based nanoarrays and stepwise sequencing were combined using Combinational Probe-Anchor Synthesis Sequencing Method and were analyzed.

Heatmap for differential genes was produced using pheatmap package of R. GO enrichment analysis and KEGG analysis for differential genes were performed in clusterProfiler package [55] version 2.2.4 of R using default settings.

### Western blotting

Tissues and cells were lysed with RIPA buffer containing Halt Protease/Phosphatase inhibitors (Thermo Fisher Scientifics, Pittsburgh, PA). The primary antibodies used in this study were as follows: anti-phospho (Ser563)-HSL (#4139), anti-phospho (Ser660)-HSL (#4126), anti-HSL (#4107), anti-pPKA Substrate (#9621), and anti-phospho (Ser133)-CREB (#9198) (all from Cell Signaling Technology); anti-SLC22A3 antibody (#ab191446), anti-ATP5A (#ab176569), anti-COX7B (#ab140629), anti-UQCRH (#ab134949), anti-UQCRB (#ab190360), and anti-beta Actin (ab8226) (all from Abcam); anti-UCP1 (#GTX112784) (Genetex); and anti-PGC1α (#20658) (Proteintech Group Inc).

### Real-time PCR analysis

Total RNA was extracted by RNeasy Mini Lipid Tissue Kit (QIAGEN, Hilden, Germany). Reverse transcription (Tiangen, Beijing, China) and SYBR green quantitative PCR (Transgen, Beijing, China) were performed according to the manufacturer’s instructions. Primers for target sequences were as shown in S2 Table. Relative gene expression level was normalized by TATA box-binding protein (Tbp) expression levels, unless otherwise indicated, since TBP expression was stable across ATs [56].

For mitochondrial DNA analysis, mouse tissues were homogenized, and genomic DNA was extracted by TIANamp genomic DNA kit (Tiangen, Beijing, China). SYBR green quantitative PCR (Transgen, Beijing, China) was performed in duplicate using mitochondrial DNA specific primers for mitochondrial Cox2 and normalized by amplification of the nuclear gene ribosomal protein s18 (Rps18).

### Lactate concentration

Lactate concentration in the ATs was measured by using a Lactate Assay Kit (Biovision, USA) following the instructions of the manufacturer.

### Seahorse experiments

Cellular OCRs were determined using a Seahorse XFe96 Extracellular Flux Analyzer (Seahorse Biosciences, Chicopee, MA). Primary adipocytes from ingWAT were seeded at 20,000 cells/well. Differentiation was induced as described above, and the cells were analyzed on day 6. Before the measurements, the cells were washed twice with assay medium (XF DMEM + 25 mM glucose + 2 mM pyruvate + 4 mM glutamine) and incubated in 175 μL of assay medium for 1 hour in an incubator without CO_2_ at 37°C. Port injection solutions were prepared as follows: NE (1 μM final concentration) or NE with propranolol (50 μM final concentration), oligomycin (2 μM final concentration), FCCP (1 μM final concentration), and a cocktail containing rotenone (1 μM) and antimycin A (1 μM). Each cycle consisted of mix 5 minutes, wait 0 minutes, and measure 5 minutes.

### Human genetic association studies for BMR

Publicly available resources were surveyed to identify human genetic studies associated with BMR. The resources included GWAS Catalog (https://www.ebi.ac.uk/gwas/home), dbGAP (https://www.ncbi.nlm.nih.gov/gap), and UK BioBank resource (http://geneatlas.roslin.ed.ac.uk/, https://www.biorxiv.org/content/early/2017/08/18/176834).

In Gene Atlas, which housed the results of all the genetic association studies analyses of the UK Biobank cohort (*N* = 408,455), BMR was available for over 7,000 participants. HaploReg version 4.1 [57] and GTEx Portal [58] were used to determine the location, consequence of the variants, and the effect of the variants on transcript levels of the gene. Information about the methods used for determining BMR of the participants were available online (http://biobank.ctsu.ox.ac.uk/crystal/docs/Anthropometry.pdf).

### HFD treatment

Six-week-old adipose-specific Oct3 KO and Ctrl mice were treated with HFD for 12 weeks under RT or thermoneutrality (30°C). For the groups under thermoneutrality, mice were then cold-stimulated at 4°C for 7 days. Body temperatures of mice were monitored at the first 8 hours under cold exposure. Body weights were recorded before and after cold exposure. Food intake was recorded daily during cold exposure. Blood was collected into EDTA tubes, and plasma was separated by centrifugation. Plasma triglyceride and total cholesterol levels were determined by respective assay kits (Nanjing Jiancheng Biotechnology Institute, Nanjing, China). Commercially available ELISA kits were used to measure plasma adiponectin (Abcam), insulin (ALPCO, Salem, NH), serum and tissue NE (Rocky Mountain Diagnostics, Colorado Springs, CO) levels following instructions from the manufacturers. Plasma FFAs was measured NEFA C kits (Wako, Oxoid SA, France). Liver triglyceride content was measured using Triglyceride Quantification Assay Kit (Abcam).

### Oral glucose tolerance test

Oral glucose tolerance test was performed on mice fed with 12-week HFD under thermoneutrality and then acclimated cold exposure for 7 days by oral gavage of glucose (2 g/kg) after 12-hour overnight fasting.

### ATMSCs

Human MSCs derived from human AT, a kind gift from Dr. Yanan Du (Tsinghua University), were cultured in human MSC growth medium (Wuhan Viraltherapy Technologies Co. Ltd, Beijing) containing 10% calf serum and penicillin G. Adipogenic differentiation of human MSCs were performed as previously described [59].

### Statistical analysis

Statistical analysis was performed using GraphPad Prism 5.0 (http://www.graphpad.com/scientific-software/prism). ANCOVA was conducted for in vivo metabolic data. Other analysis was conducted by Student *t* test (for comparison of two experimental conditions) or one-way ANOVA followed by Tukey's test (for comparison of three or more experimental conditions). Data are represented as mean ± standard deviation. Statistical significance was calculated and indicated (*p < 0.05, **p < 0.01, ***p < 0.001).

## Supporting information

S1 Fig Oct3 was highly expressed in AT. (A) Tissue distribution of human *OCT3* mRNA (*n* = 3). (B) mRNA expression of *Perilipin* in mouse MAs and SVCs of ingWAT (*n* = 3). (C) mRNA expression of *Oct3* in mouse MA and SVC of gonWAT (*n* = 3). (D) mRNA expression of *Perilipin* in mouse MA and SVC of gonWAT (*n* = 3). (E) Three-dimensional–structure modeling of Oct3 and molecular docking of different monoamines. The three-dimensional Oct3 homology model was based on human GLUT3 template in the outward-facing-occluded (“occluded”) conformation in complex with D-glucose. The predicted homology models contained the whole target sequence including the 12 transmembrane helices and the primary substrate binding site. After overall structural quality evaluation with DOPE scores, the best-scored model was further assessed based on its ability to discriminate between catecholamines (NE, epinephrine, histamine, dopamine, serotonin, and tyramine) and d-AMPH, which was not a substrate for Oct3 and only exerted weak inhibitory effects on Oct3-mediated uptake. The numerical data underlying this figure are included in S1 Data. AT, adipose tissue; d-AMPH, dextroamphetamine; DOPE, discrete optimized protein energy; GLUT3, glucose transporter 3; gonWAT, gonadal white adipose tissue; ingWAT, inguinal white adipose tissue; MA, mature adipocyte; NE, norepinephrine; Oct3, organic cation transporter 3; SVC, stromal vascular cell.
(TIF)

S2 Fig Characterization of adipose-specific *Oct3* KO (cKO) mice. (A) Schematic diagram of the construction of cKO mice. *Oct3*^*fl/fl*^ mice were generated by locating LoxP sites *in cis* flanking in exon 2 of the *Oct3* allele. (B) Western blot analysis of Oct3 in multiple ATs from Ctrl and cKO mice. (C) Analysis of gene expression by real-time PCR in ingWAT and gonWAT from Ctrl and cKO mice (*n* = 6). (D) Body weight of Ctrl and cKO mice. (E–H) Metabolic parameters of Ctrl and cKO mice under RT (*n* = 4). (E) O_2_ consumption; (F) CO_2_ production; (G) heat production; (H) RER. (I) Representative HE staining in BAT, ingWAT and gonWAT (*n* = 3–4). Scale bar, 100 μm. Data in C–D were analyzed by Student *t* test. Data in E–H were analyzed by ANCOVA analysis. The numerical data underlying this figure are included in S1 Data. AT, adipose tissue; cKO, conditional knockout; Ctrl, control; gonWAT, gonadal white adipose tissue; HE, hematoxylin–eosin; ingWAT, inguinal white adipose tissue; RER, respiratory exchange ratio; RT, room temperature; Oct3, organic cation transporter 3.
(TIF)

S3 Fig In vivo NE uptake and analysis of CLAMS by ANCOVA. (A) Experimental scheme of in vivo NE uptake assay in AT from Ctrl and cKO mice. (B) Representative figures of ingWAT immunolabeled by anti-tyrosine hydroxylase (Th) to verify successful denervation (when ingWAT was successfully denervated, Th, a sympathetic nerve marker, would be significantly decreased). (C) Body composition of Ctrl and cKO mice. The total mass of mice consists of lean mass and fat mass (*n* = 8). (D, E) Multiple linear regression model and ANCOVA analysis for coefficient estimates of oxygen consumption (panel D) and energy expenditure (panel E) to lean mass and fat mass in Ctrl and cKO mice (*n* = 8). (F, G) The relationship of oxygen consumption (panel F) and energy expenditure (panel G) to body weight (*n* = 8). Data in S3D–S3G Fig were analyzed by ANCOVA to determine statistical differences. The numerical data underlying this figure are included in S1 Data. AT, adipose tissue; cKO, conditional knockout; CLAMS, Comprehensive Lab Animal Monitoring System; Ctrl, control; ingWAT, inguinal white adipose tissue; NE, norepinephrine.
(TIF)

S4 Fig Thermogenic and lipolytic response in BAT of Ctrl and cKO mice after NE stimulation. (A) mRNA expression of thermogenic and lipolytic genes in BAT of Ctrl and *c*KO mice after NE injection (*n* = 3–4). (B) Basal and epinephrine-stimulated serum FFA in Ctrl and *c*KO mice (*n* = 6). (C) Protein levels of pHsl-S563, pHsl-S660, and total Hsl in BAT of Ctrl and *c*KO mice with NE (“NE”) or without NE injection (Ctrl). (D) Oil red O staining of 3T3-L1 cells stably transfected with EVs or *Oct3* (*Oct3*-OE) induced by differentiation medium. (E) Basal and forskolin-stimulated lipolysis, as measured by glycerol release from differentiated 3T3-L1 adipocytes stably transfected with EVs or Oct3 (Oct3-OE) (*n* = 3). (F) Representative micrographs of livers of Ctrl and cKO mice with NE (“NE”) and without NE injection (Saline), stained with HE. (G) Hepatic triglyceride levels of Ctrl and cKO mice with NE (“NE”) and without NE injection (Saline) (*n* = 6). Data in panels A, B, E, and G were analyzed by Student *t* test. The numerical data underlying this figure are included in S1 Data. BAT, brown adipose tissue; cKO, conditional knockout; Ctrl, control; EV, empty vector; FFA, free fatty acid; NE, norepinephrine; Oct3, organic cation transporter 3; pHsl, phosphorylated hormone-sensitive lipase.
(TIF)

S5 Fig Thermogenic response in BAT and gonWAT after cold challenge. Ctrl and cKO mice were housed at 4°C for 1 month. (A) Body weights of Ctrl and cKO mice (*n* = 6). (B, C) NE content in ingWAT (panel B) and serum (panel C) from Ctrl and cKO mice after cold exposure (*n* = 6). (D) Heat maps of significantly changed gene expressions in ingWAT of Ctrl and cKO mice. (E) Representative HE staining in BAT and gonWAT. Scale bar, 100 μm. (F) mRNA expression of thermogenic genes, catecholamine degradation enzymes, and FAO genes in BAT (*n* = 4). (G) mRNA expression of thermogenic, WAT-selective and FAO genes in gonWAT (*n* = 4). (H, I) Protein levels of pCreb, total Creb, Ucp1, and Pgc1α in BAT (panel H) and gonWAT (panel I). (J) mRNA expression of cAMP-PKA pathway component genes in ingWAT (*n* = 4). (K, L) mRNA expression of ETC and glycolytic genes in BAT (panel K) and gonWAT (panel L) (*n* = 4). (M) The amount of lactate in BAT and gonWAT. Data in A–C, F–G, and J–M were analyzed by Student *t* test. The numerical data underlying this figure are included in S1 Data. BAT, brown adipose tissue; cKO, conditional knockout; Ctrl, control; ETC, electron transport chain; FAO, fatty acid oxidation; gonWAT, gonadal white adipose tissue; ingWAT, inguinal white adipose tissue; NE, norepinephrine; PKA, protein kinase A.
(TIF)

S6 Fig Characterization of *Oct3 knockout* primary inguinal adipocytes. (A) OCR measured by Seahorse in differentiated primary inguinal adipocytes from Ctrl and cKO mice (*n* = 6). (B) *Ap2* mRNA expression in differentiated primary inguinal adipocytes from Ctrl and cKO mice after stimulation with NE and T_3_ in the presence or absence of propranolol (*n* = 3). (C) *Ucp1* mRNA expression in differentiated primary inguinal adipocytes from Ctrl and cKO mice after stimulation with epinephrine, dopamine, and serotonin in the presence of T_3_ (*n* = 3). (D) Western blotting of pCreb and total Creb in primary inguinal adipocytes from Ctrl and cKO stimulated with epinephrine, dopamine, and serotonin in the presence of T_3._ Data in A–C were analyzed by Student *t* test. The numerical data underlying this figure are included in S1 Data. cKO, conditional knockout; Ctrl, control; NE, norepinephrine; OCR, O_2_ consumption rate; Oct3, organic cation transporter 3; pCreb, phosphorylated cAMP-responsive element binding protein; T_3_, triiodothyronine.
(TIF)

S7 Fig Effect of Oct3 deficiency on HFD-fed mice under cold exposure. (A) Body weight curves of Ctrl and cKO mice fed HFD for 12 weeks at thermoneutrality (30°C) (panel A) or RT (panel B) (thermoneutrality, *n* = 8; RT, *n* = 6). (C–L) Ctrl and cKO mice in panel A were then subjected to a cold challenge (4°C) for 1 week (*n* = 8). (C) Body temperature. (D) Body weights. (E) Food intake. (F) Fed blood glucose. (G) Plasma triglyceride. (H) Plasma cholesterol. (I) Plasma insulin. (J) Plasma adiponectin. (K) Plasma FFAs. (L) Glucose tolerance test. All data were analyzed by Student *t* test. The numerical data underlying this figure are included in S1 Data. cKO, conditional knockout; Ctrl, control; FFA, free fatty acid; HFD, high-fat diet; Oct3, organic cation transporter 3; RT, room temperature.
(TIF)

S8 Fig Polymorphisms in human *SLC22A3* and their associations with BMR and transcript levels. (A) A regional plot of *SLC22A3* locus. SNPs were plotted by chromosome 6 against association with BMR in UK Biobank participants (*N* ~ 7,000). An SNP, rs555754 (purple circle), and its proxies are the top signals in *SLC22A3* locus. Estimated recombination rates (cM/Mb) were plotted in blue to reflect the local LD structure. The SNPs surrounding the significant SNP, rs555754, were color coded to reflect their LD with this SNP. This LD was taken from pairwise r^2^ values from the 1000 Genomes Nov 2014 EUR (hg19). Genes, the position of exons, and the direction of the transcription from the UCSC Genome Browser were noted. This plot was created using LocusZoom (http://locuszoom.org/genform.php?type=yourdata). The associations for each variant in this plot (effect size of the reference allele and *p*-value) were shown in S3 Table. (B, C) *SLC22A3* transcript levels in adipose subcutaneous (panel B) and adipose visceral omentum (panel C) were significantly associated with the top two SNPs in the locus zoom plot in panel A. The figure and data were available in GTEx portal (gtexportal.org). The x-axis showed the genotype for the SNP and the y-axis showed the expression levels of *SLC22A3* as quantified by RNA-seq method. A full list of all eQTL for each SNPs were available in S3 Table. (D) mRNA expression level of catecholamine transporters and catecholamine degradation enzymes in human AT (*n* = 3). (E) Oil Red O staining of undifferentiated and adipogenically differentiated ATMSC. (F) mRNA expression level of catecholamine transporters and catecholamine degradation enzymes in differentiated ATMSC (*n* = 3). (G) In vitro glycerol release from differentiated ATMSC untreated or NE-treated to stimulate lipolysis, incubated with vehicle or OCT3 inhibitor decynium-22 (10 μM) (*n* = 3). Data in panel G were analyzed by Student *t* test. The numerical data underlying this figure are included in S1 Data. AT, adipose tissue; ATMSC, AT-derived mesenchymal stem cell; BMR, basal metabolic rate; eQTL, expression quantitative trait locus; LD, linkage disequilibrium; NE, norepinephrine; Oct3, organic cation transporter 3; SNP, single-nucleotide polymorphism.
(TIF)

S1 Table RNA-seq data summary. (XLSX)

S2 Table Primers used for real-time PCR. (XLSX)

S3 Table Genetic associations of variants in SLC22A3 with basal metabolic rate. (XLSX)

S1 Data Numerical values of presented diagrams. (XLSX)

## Abbreviations

β-AR: β-adrenergic receptor
AT: adipose tissue
ATMSC: AT-derived mesenchymal stem cell
AU: arbitrary unit
BAT: brown adipose tissue
BMR: basal metabolic rate
cAMP: cyclic adenosine monophosphate
cKO: conditional knockout
CLAMS: Comprehensive Lab Animal Monitoring System
Cox2: cytochrome c oxidase subunit 2
Creb: cAMP-responsive element binding protein
dbGAP: the database of Genotypes and Phenotypes
DIO: diet-induced obesity
DNB: DNA nanoball
DOPE: discrete optimized protein energy
ETC: electron transport chain
EV: empty vector
FAO: fatty acid oxidation
FFA: free fatty acid
GO: gene ontology
gonWAT: gonadal white adipose tissue
G_s_: G_s_ alpha subunit
HE: hematoxylin–eosin
HFD: high-fat diet
hGLUT3: human glucose transporter 3
Hsl: hormone-sensitive lipase
ingWAT: inguinal white adipose tissue
KEGG: Kyoto Encyclopedia of Genes and Genomes
KO: knockout
MAO: monoamine oxidase
MPP: methyl-4-phenylpyridinium
MSC: mesenchymal stem cell
MUSCLE: Multiple Sequence Comparison by Log-Expectation
NE: norepinephrine
NET: norepinephrine transporter
OCR: O_2_ consumption rate
Oct3: organic cation transporter 3
pCreb: phosphor-Creb
Pdk4: pyruvate dehydrogenase kinase 4
PKA: cAMP-dependent protein kinase A
pPKA: phospho-PKA
RCR: rolling circle replication
RNA-seq: RNA sequencing
Rps18: ribosomal protein s18
RT: room temperature
SNP: single-nucleotide polymorphism
ssDNA: single-strand DNA
SVC: stromal vascular cell
SVF: stromal vascular fraction
T_3_: triiodothyronine
Tbp: TATA box-binding protein
TEM: transmission electron microscope
TF: transcription factor
Ucp: uncoupling protein
VO_2_: oxygen consumption
WAT: white adipose tissue
