# Supplementary figures and images for: Organic cation transporter 3 (Oct3) is a distinct catecholamines clearance route in adipocytes mediating the beiging of white adipose tissue
Source: PLoS Biol. 2019 Jan 17;17(1):e2006571. doi: 10.1371/journal.pbio.2006571 (PMC6336244; doi:10.1371/journal.pbio.2006571)

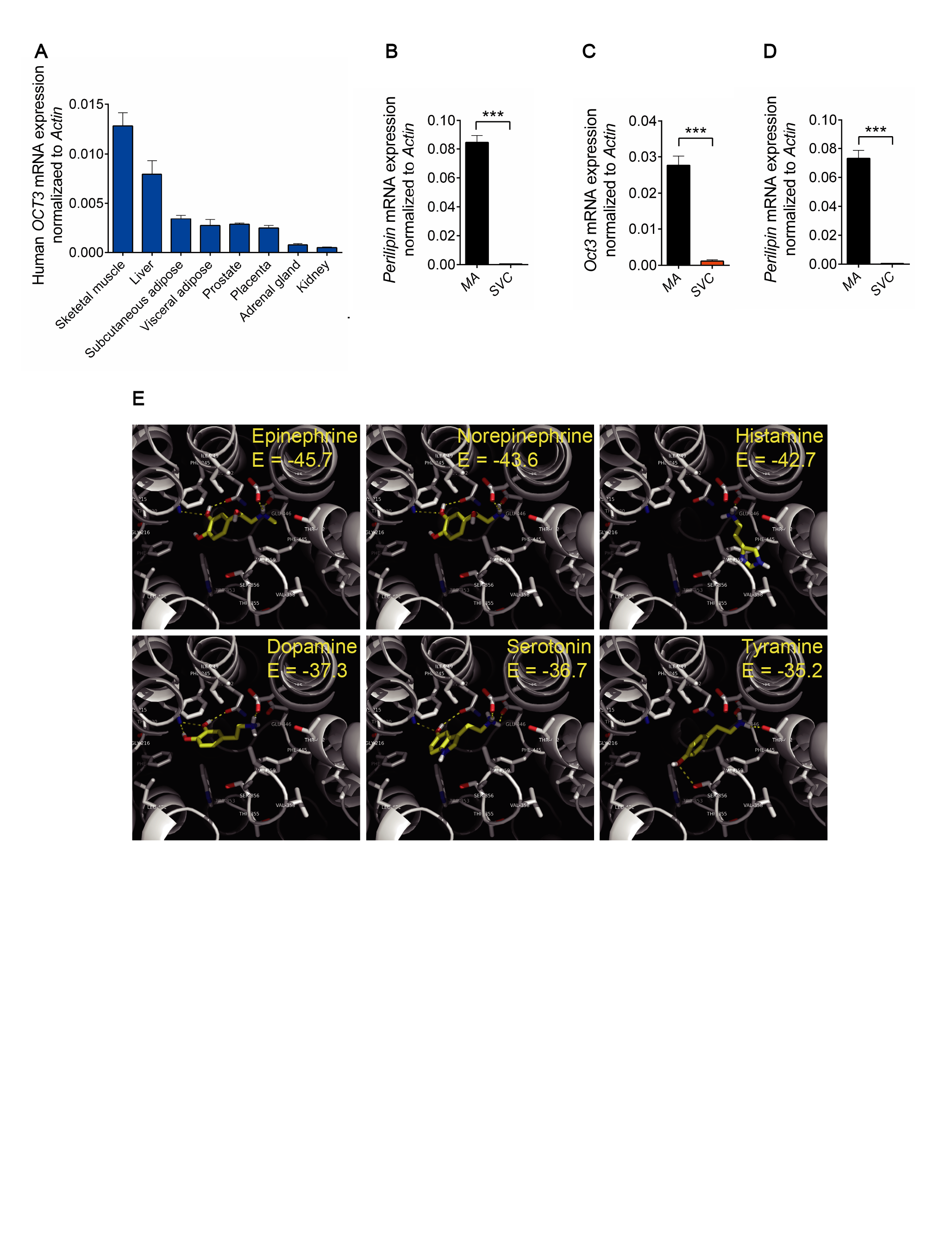

Supplement: S1 Fig — (A) Tissue distribution of human OCT3 mRNA (n = 3). (B) mRNA expression of Perilipin in mouse MAs and SVCs of ingWAT (n = 3). (C) mRNA expression of Oct3 in mouse MA and SVC of gonWAT (n = 3). (D) mRNA expression of Perilipin in mouse MA and SVC of gonWAT (n = 3). (E) Three-dimensional–structure modeling of Oct3 and molecular docking of different monoamines. The three-dimensional Oct3 homology model was based on human GLUT3 template in the outward-facing-occluded (“occluded”) conformation in complex with D-glucose. The predicted homology models contained the whole target sequence including the 12 transmembrane helices and the primary substrate binding site. After overall structural quality evaluation with DOPE scores, the best-scored model was further assessed based on its ability to discriminate between catecholamines (NE, epinephrine, histamine, dopamine, serotonin, and tyramine) and d-AMPH, which was not a substrate for Oct3 and only exerted weak inhibitory effects on Oct3-mediated uptake. The numerical data underlying this figure are included in S1 Data. AT, adipose tissue; d-AMPH, dextroamphetamine; DOPE, discrete optimized protein energy; GLUT3, glucose transporter 3; gonWAT, gonadal white adipose tissue; ingWAT, inguinal white adipose tissue; MA, mature adipocyte; NE, norepinephrine; Oct3, organic cation transporter 3; SVC, stromal vascular cell. (TIF) [file pbio.2006571.s001.tif]

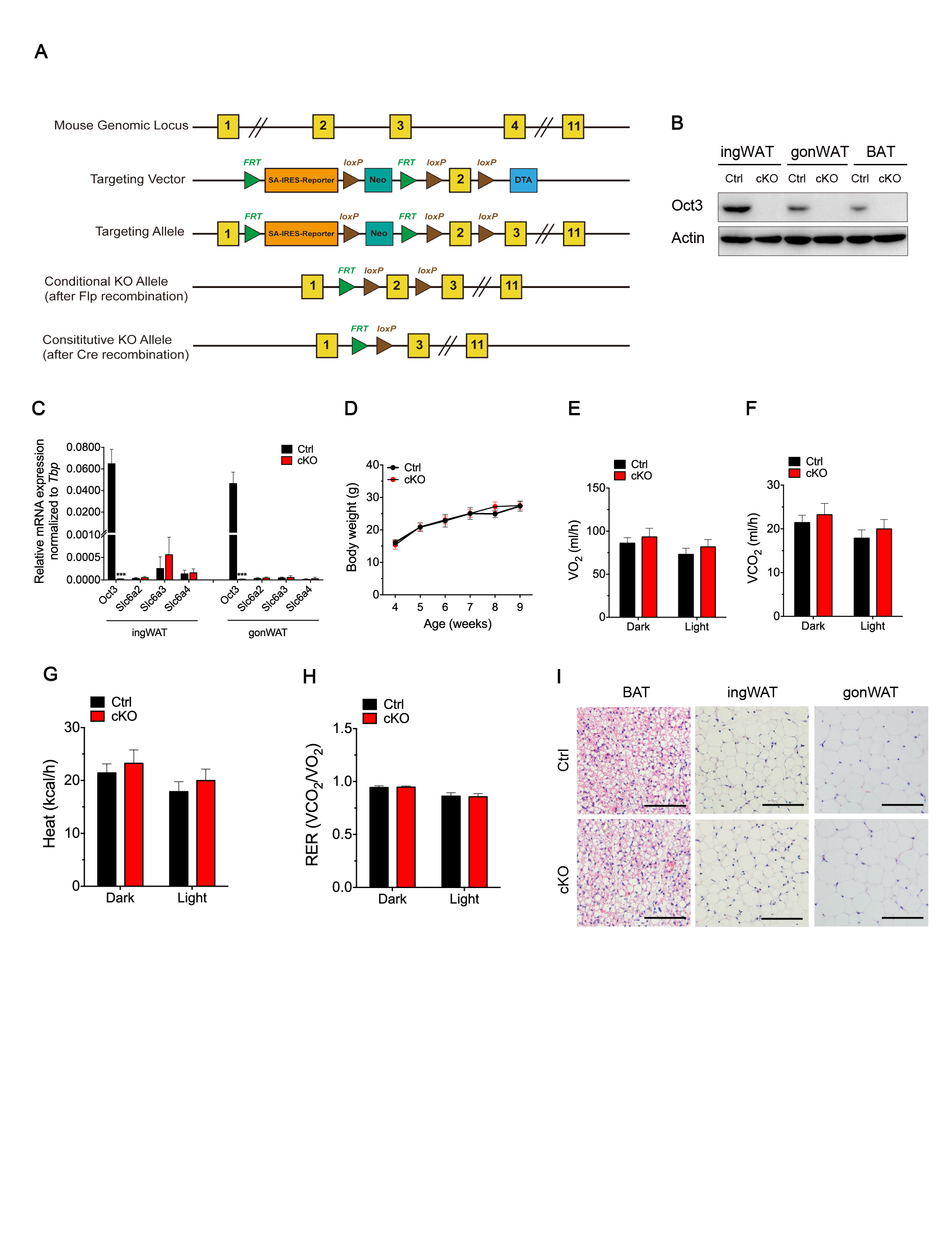

Supplement: S2 Fig — (A) Schematic diagram of the construction of cKO mice. Oct3fl/fl mice were generated by locating LoxP sites in cis flanking in exon 2 of the Oct3 allele. (B) Western blot analysis of Oct3 in multiple ATs from Ctrl and cKO mice. (C) Analysis of gene expression by real-time PCR in ingWAT and gonWAT from Ctrl and cKO mice (n = 6). (D) Body weight of Ctrl and cKO mice. (E–H) Metabolic parameters of Ctrl and cKO mice under RT (n = 4). (E) O2 consumption; (F) CO2 production; (G) heat production; (H) RER. (I) Representative HE staining in BAT, ingWAT and gonWAT (n = 3–4). Scale bar, 100 μm. Data in C–D were analyzed by Student t test. Data in E–H were analyzed by ANCOVA analysis. The numerical data underlying this figure are included in S1 Data. AT, adipose tissue; cKO, conditional knockout; Ctrl, control; gonWAT, gonadal white adipose tissue; HE, hematoxylin–eosin; ingWAT, inguinal white adipose tissue; RER, respiratory exchange ratio; RT, room temperature; Oct3, organic cation transporter 3. (TIF) [file pbio.2006571.s002.tif]

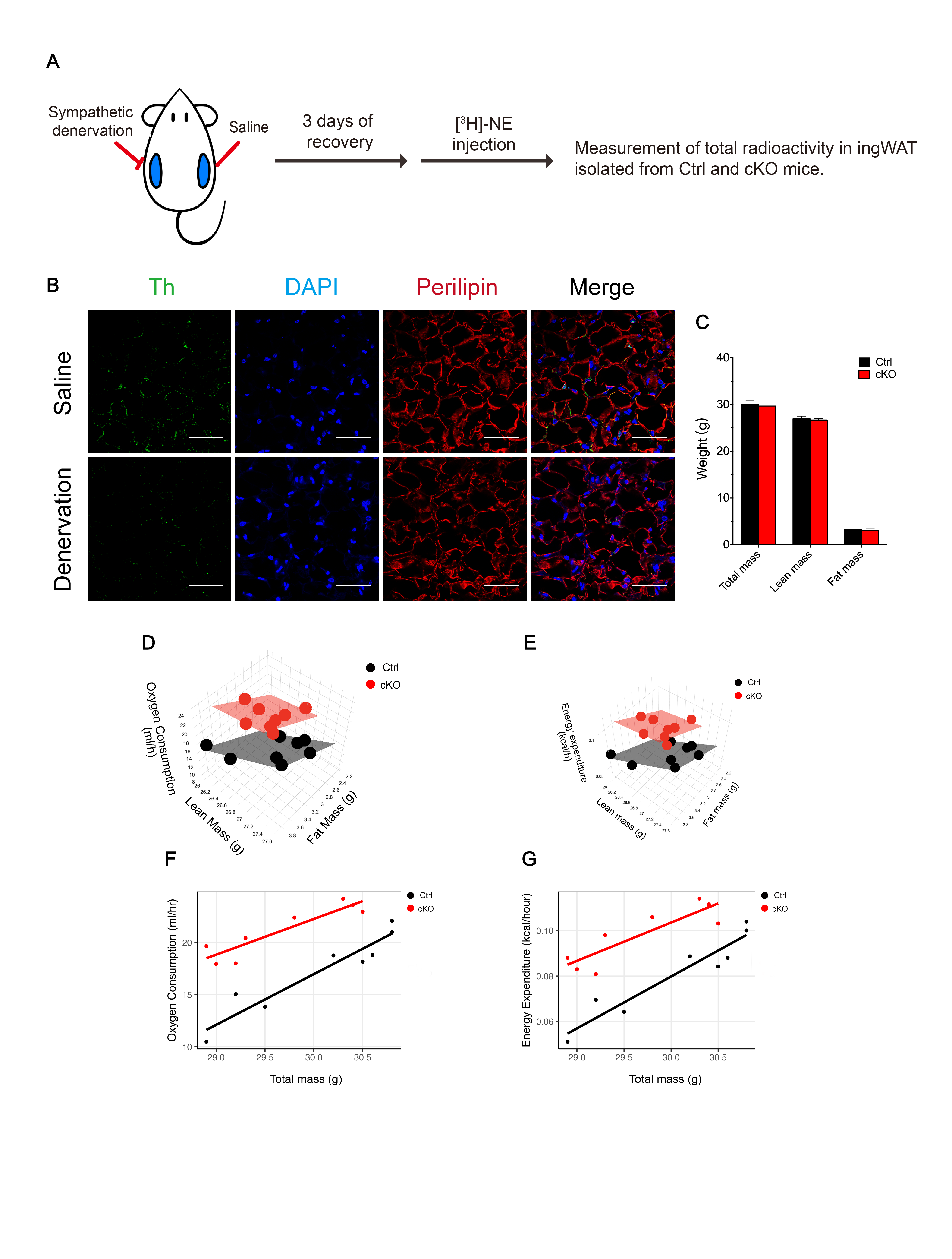

Supplement: S3 Fig — (A) Experimental scheme of in vivo NE uptake assay in AT from Ctrl and cKO mice. (B) Representative figures of ingWAT immunolabeled by anti-tyrosine hydroxylase (Th) to verify successful denervation (when ingWAT was successfully denervated, Th, a sympathetic nerve marker, would be significantly decreased). (C) Body composition of Ctrl and cKO mice. The total mass of mice consists of lean mass and fat mass (n = 8). (D, E) Multiple linear regression model and ANCOVA analysis for coefficient estimates of oxygen consumption (panel D) and energy expenditure (panel E) to lean mass and fat mass in Ctrl and cKO mice (n = 8). (F, G) The relationship of oxygen consumption (panel F) and energy expenditure (panel G) to body weight (n = 8). Data in S3D–S3G Fig were analyzed by ANCOVA to determine statistical differences. The numerical data underlying this figure are included in S1 Data. AT, adipose tissue; cKO, conditional knockout; CLAMS, Comprehensive Lab Animal Monitoring System; Ctrl, control; ingWAT, inguinal white adipose tissue; NE, norepinephrine. (TIF) [file pbio.2006571.s003.tif]

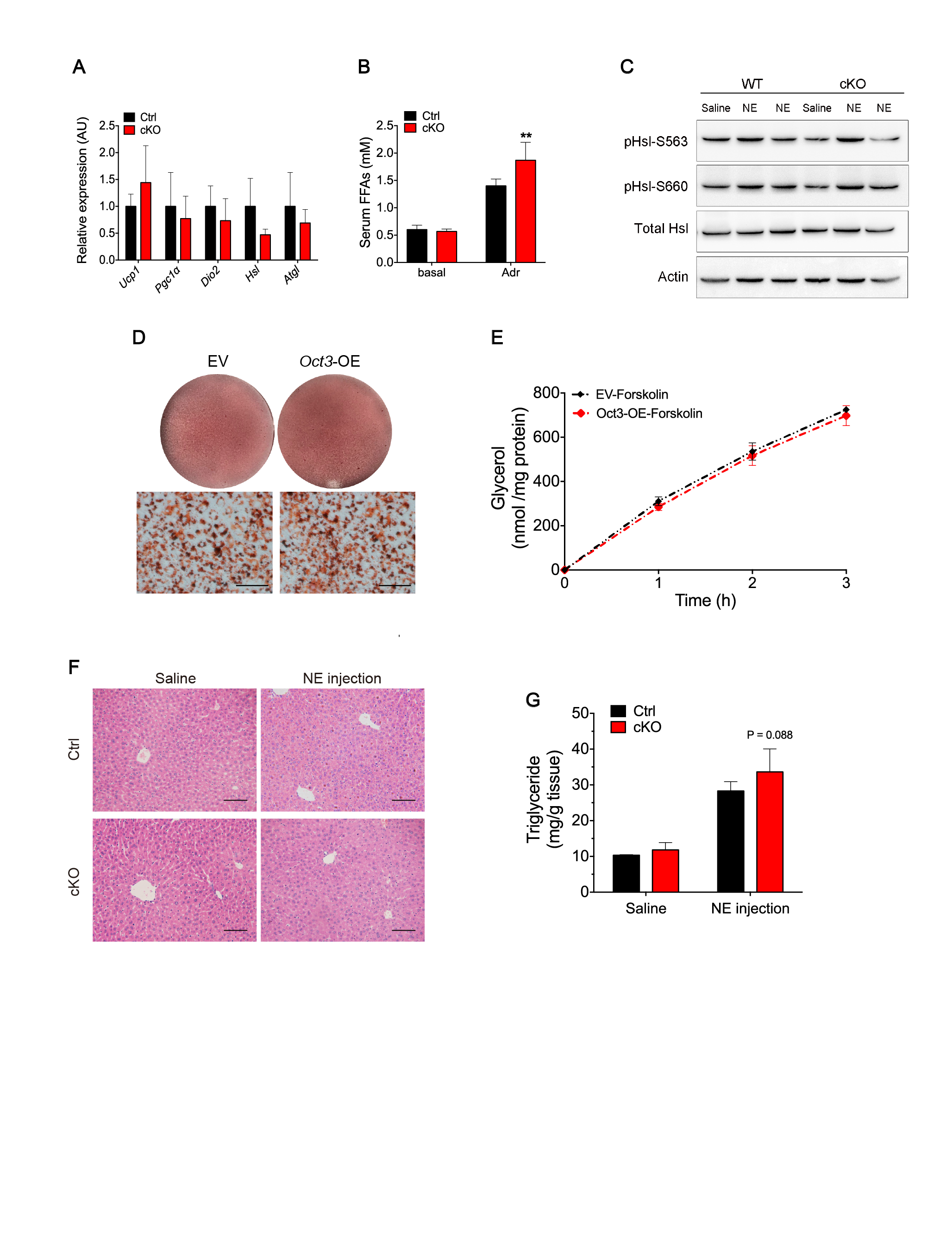

Supplement: S4 Fig — (A) mRNA expression of thermogenic and lipolytic genes in BAT of Ctrl and cKO mice after NE injection (n = 3–4). (B) Basal and epinephrine-stimulated serum FFA in Ctrl and cKO mice (n = 6). (C) Protein levels of pHsl-S563, pHsl-S660, and total Hsl in BAT of Ctrl and cKO mice with NE (“NE”) or without NE injection (Ctrl). (D) Oil red O staining of 3T3-L1 cells stably transfected with EVs or Oct3 (Oct3-OE) induced by differentiation medium. (E) Basal and forskolin-stimulated lipolysis, as measured by glycerol release from differentiated 3T3-L1 adipocytes stably transfected with EVs or Oct3 (Oct3-OE) (n = 3). (F) Representative micrographs of livers of Ctrl and cKO mice with NE (“NE”) and without NE injection (Saline), stained with HE. (G) Hepatic triglyceride levels of Ctrl and cKO mice with NE (“NE”) and without NE injection (Saline) (n = 6). Data in panels A, B, E, and G were analyzed by Student t test. The numerical data underlying this figure are included in S1 Data. BAT, brown adipose tissue; cKO, conditional knockout; Ctrl, control; EV, empty vector; FFA, free fatty acid; NE, norepinephrine; Oct3, organic cation transporter 3; pHsl, phosphorylated hormone-sensitive lipase. (TIF) [file pbio.2006571.s004.tif]

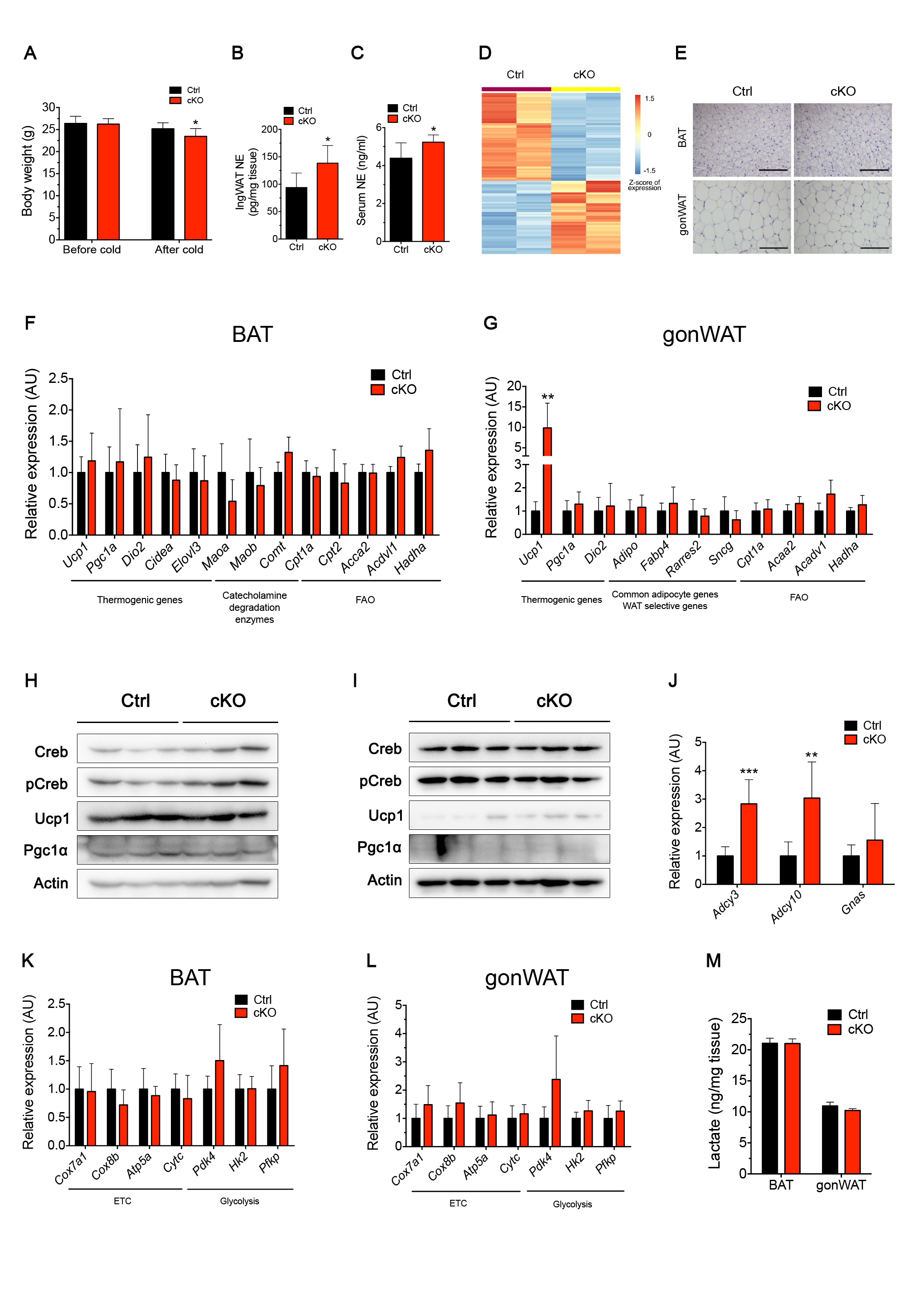

Supplement: S5 Fig — Ctrl and cKO mice were housed at 4°C for 1 month. (A) Body weights of Ctrl and cKO mice (n = 6). (B, C) NE content in ingWAT (panel B) and serum (panel C) from Ctrl and cKO mice after cold exposure (n = 6). (D) Heat maps of significantly changed gene expressions in ingWAT of Ctrl and cKO mice. (E) Representative HE staining in BAT and gonWAT. Scale bar, 100 μm. (F) mRNA expression of thermogenic genes, catecholamine degradation enzymes, and FAO genes in BAT (n = 4). (G) mRNA expression of thermogenic, WAT-selective and FAO genes in gonWAT (n = 4). (H, I) Protein levels of pCreb, total Creb, Ucp1, and Pgc1α in BAT (panel H) and gonWAT (panel I). (J) mRNA expression of cAMP-PKA pathway component genes in ingWAT (n = 4). (K, L) mRNA expression of ETC and glycolytic genes in BAT (panel K) and gonWAT (panel L) (n = 4). (M) The amount of lactate in BAT and gonWAT. Data in A–C, F–G, and J–M were analyzed by Student t test. The numerical data underlying this figure are included in S1 Data. BAT, brown adipose tissue; cKO, conditional knockout; Ctrl, control; ETC, electron transport chain; FAO, fatty acid oxidation; gonWAT, gonadal white adipose tissue; ingWAT, inguinal white adipose tissue; NE, norepinephrine; PKA, protein kinase A. (TIF) [file pbio.2006571.s005.tif]

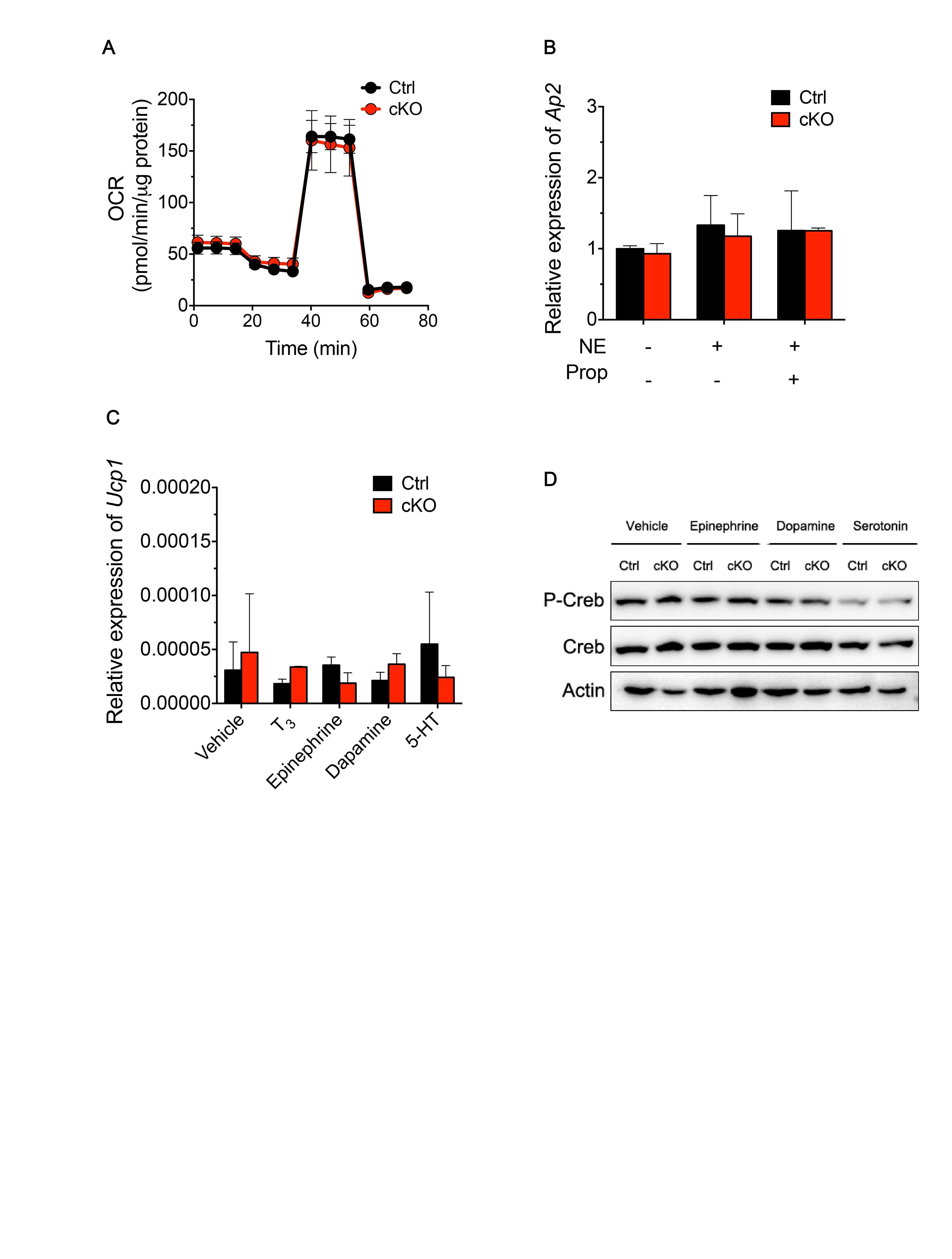

Supplement: S6 Fig — (A) OCR measured by Seahorse in differentiated primary inguinal adipocytes from Ctrl and cKO mice (n = 6). (B) Ap2 mRNA expression in differentiated primary inguinal adipocytes from Ctrl and cKO mice after stimulation with NE and T3 in the presence or absence of propranolol (n = 3). (C) Ucp1 mRNA expression in differentiated primary inguinal adipocytes from Ctrl and cKO mice after stimulation with epinephrine, dopamine, and serotonin in the presence of T3 (n = 3). (D) Western blotting of pCreb and total Creb in primary inguinal adipocytes from Ctrl and cKO stimulated with epinephrine, dopamine, and serotonin in the presence of T3. Data in A–C were analyzed by Student t test. The numerical data underlying this figure are included in S1 Data. cKO, conditional knockout; Ctrl, control; NE, norepinephrine; OCR, O2 consumption rate; Oct3, organic cation transporter 3; pCreb, phosphorylated cAMP-responsive element binding protein; T3, triiodothyronine. (TIF) [file pbio.2006571.s006.tif]

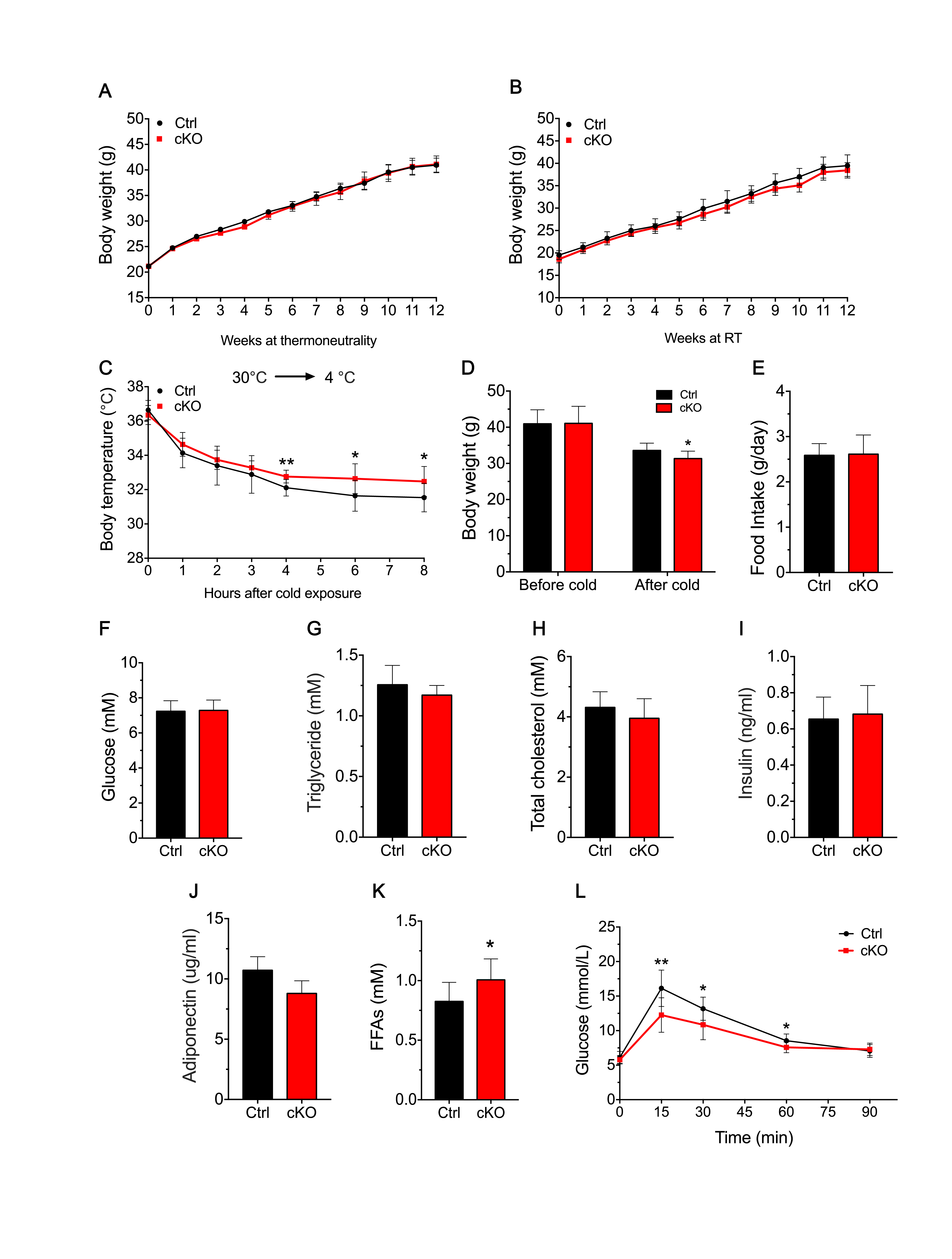

Supplement: S7 Fig — (A) Body weight curves of Ctrl and cKO mice fed HFD for 12 weeks at thermoneutrality (30°C) (panel A) or RT (panel B) (thermoneutrality, n = 8; RT, n = 6). (C–L) Ctrl and cKO mice in panel A were then subjected to a cold challenge (4°C) for 1 week (n = 8). (C) Body temperature. (D) Body weights. (E) Food intake. (F) Fed blood glucose. (G) Plasma triglyceride. (H) Plasma cholesterol. (I) Plasma insulin. (J) Plasma adiponectin. (K) Plasma FFAs. (L) Glucose tolerance test. All data were analyzed by Student t test. The numerical data underlying this figure are included in S1 Data. cKO, conditional knockout; Ctrl, control; FFA, free fatty acid; HFD, high-fat diet; Oct3, organic cation transporter 3; RT, room temperature. (TIF) [file pbio.2006571.s007.tif]

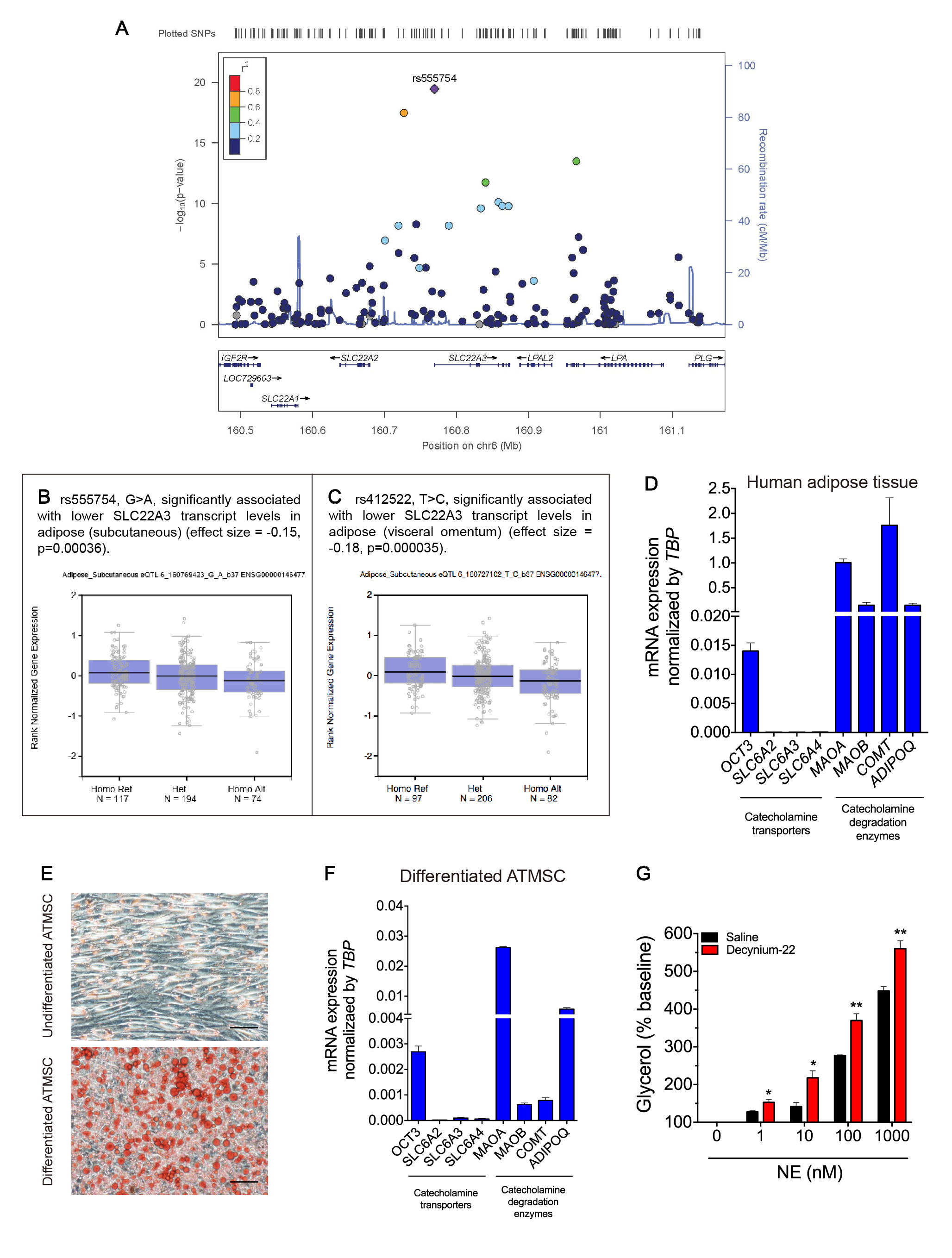

Supplement: S8 Fig — (A) A regional plot of SLC22A3 locus. SNPs were plotted by chromosome 6 against association with BMR in UK Biobank participants (N ~ 7,000). An SNP, rs555754 (purple circle), and its proxies are the top signals in SLC22A3 locus. Estimated recombination rates (cM/Mb) were plotted in blue to reflect the local LD structure. The SNPs surrounding the significant SNP, rs555754, were color coded to reflect their LD with this SNP. This LD was taken from pairwise r2 values from the 1000 Genomes Nov 2014 EUR (hg19). Genes, the position of exons, and the direction of the transcription from the UCSC Genome Browser were noted. This plot was created using LocusZoom (http://locuszoom.org/genform.php?type=yourdata). The associations for each variant in this plot (effect size of the reference allele and p-value) were shown in S3 Table. (B, C) SLC22A3 transcript levels in adipose subcutaneous (panel B) and adipose visceral omentum (panel C) were significantly associated with the top two SNPs in the locus zoom plot in panel A. The figure and data were available in GTEx portal (gtexportal.org). The x-axis showed the genotype for the SNP and the y-axis showed the expression levels of SLC22A3 as quantified by RNA-seq method. A full list of all eQTL for each SNPs were available in S3 Table. (D) mRNA expression level of catecholamine transporters and catecholamine degradation enzymes in human AT (n = 3). (E) Oil Red O staining of undifferentiated and adipogenically differentiated ATMSC. (F) mRNA expression level of catecholamine transporters and catecholamine degradation enzymes in differentiated ATMSC (n = 3). (G) In vitro glycerol release from differentiated ATMSC untreated or NE-treated to stimulate lipolysis, incubated with vehicle or OCT3 inhibitor decynium-22 (10 μM) (n = 3). Data in panel G were analyzed by Student t test. The numerical data underlying this figure are included in S1 Data. AT, adipose tissue; ATMSC, AT-derived mesenchymal stem cell; BMR, basal metabolic rate; eQTL, e [file pbio.2006571.s008.tif]
